# The impact of rivers and lakes on urban transportation expansion: A case study of the century-long evolution of the road network in Wuhan, China

**DOI:** 10.1371/journal.pone.0298678

**Published:** 2024-03-18

**Authors:** Ran Peng, Keyuan Ding, Haixu Guo, Xueliang Liu, Yehao Liu, Huaiyang Weng, Rui Li

**Affiliations:** 1 School of Transportation and Logistics Engineering, Wuhan University of Technology, Wuhan, Hubei, China; 2 School of Civil Engineering and Architecture, Wuhan Institute of Technology, Wuhan, Hubei, China; 3 School of Civil Engineering and Architecture, Wuhan University of Technology, Wuhan, Hubei, China; Huazhong University of Science and Technology, CHINA

## Abstract

Throughout history, rivers and lakes have wielded a profound influence on the dynamics of urban transportation expansion. To illustrate this phenomenon, we turn to the century-long evolution of the road network in Wuhan, China, as a case study. The study aims to explore the relationship framework between water bodies and urban transportation, characterized by the sequence of “strong connection” to “weakened connection”, then to “mutual restriction”, and ultimately to “mutual benefit”. Additionally, the analysis of the impact mechanisms of rivers and lakes on urban transportation at different stages of development is also a key research objective. To facilitate our exploration, we select the road networks in Wuhan from four years of 1922, 1969, 1995, and 2023 as the primary research subjects. By establishing water buffers, we scrutinize the evolving characteristics of riverside and lakeside transportation amidst the city’s expansion. Based on the modified shortest path model, we introduce the innovative concepts of “Detour Index” and “Weighted Detour Index” to assess the road accessibility of each node in the city based on its inherent environmental conditions. This allows for the effective analysis of the potential impact of water bodies as “obstacles” on the road network at different stages of urban development. The study found that in the areas adjacent to the rivers and lakes in Wuhan, there is insufficient road accessibility based on their inherent environmental conditions. Particularly, some areas along the rivers may become “terminals” in the urban road network. Furthermore, during the process of urban expansion, the correlation between the urban road network and rivers continues to weaken, while the correlation with lakes continues to strengthen. These conclusions can provide valuable insights for the planning of urban roads near water bodies.

## Introduction

Cities are large residential areas formed by the agglomeration of non-agricultural industries and non-agricultural populations. Due to the demand for drinking water and the convenience of water transportation, cities have been built near water since ancient times, and urban development and expansion have always been restricted by water resources. The extension of road is the prerequisite for urban expansion, therefore, in the process of urban expansion, there is always a certain contradiction between urban transportation and water bodies: on the one hand, the obstruction of water bodies such as rivers and lakes will become an “obstacle” to the extension of urban road network; on the other hand, the extension of urban road network will also cause damage to water bodies, thereby destroying the urban ecological environment. In ancient society, there was a strong correlation between the urban transportation and water bodies, in modern times, due to the large-scale expansion of many cities in the world, the correlation between transportation and water bodies in newly built urban areas has been greatly weakened. However, the overall urban road network pattern is still formed in the early stage of the city and continues to this day, which also shows the specificity of being affected by the local water bodies, thus showing different urban road network characteristics when the city’s water bodies is different.

In recent years, the rapid urban expansion in Chinese cities has given rise to increasingly severe transportation challenges. This study focuses on Wuhan, the capital of Hubei Province, China, a city characterized by its abundant rivers and dense lakes. Our research aims to investigate the influence of these natural water bodies on urban transportation at various stages of urban development. Additionally, we seek to analyze the historical evolution of the relationship between the urban road network and urban water bodies. The primary objective of this research is to establish a foundation for enhancing the scientific and rational aspects of urban road planning. Furthermore, our study intends to provide theoretical insights and empirical guidance for the optimization of the urban road infrastructure while ensuring the sustainable utilization of urban water resources. For this purpose, we selected standard maps of Wuhan from the years 1922, 1969, 1995, and 2023 as the research objects. We extracted vector data of road networks and water bodies from them and then used ArcGIS to create water body buffers. This enabled us to analyze the changing trends in the correlation between transportation and water bodies in Wuhan during different historical stages. We also proposed an urban regional accessibility model based on road distance/spatial distance matrices to analyze the distribution characteristics and evolutionary patterns of urban regional accessibility when water bodies are considered as “obstacles”. The main contribution of this method lies in fully considering the influence of the own environmental conditions of the road accessibility assessment objects. In comparison to traditional accessibility assessment models, which primarily focus on the location superiority of the objects, this method is more suitable for road accessibility assessments under the influence of natural geographic environments such as urban water bodies. Therefore, it possesses general applicability in similar studies.

The remainder of this paper is organized as follows: the literature review related to this study is presented in the part of Literature review; the part of Objects and methods elaborates on the research object and research methods; the part of Results and discussion presents and discusses the research results; the part of Conclusions, deficiencies, and prospects presents the conclusions, limitations, and prospects for the follow-up work of this study.

## Literature review

The presence of water bodies influences road orientation, road network layout, and the transportation behavior of the population, thereby impacting urban and regional development and planning. A few researchers have recognized this issue, such as Statzner et al. [1], who calculated the ratio of road and river curvature in different natural environments like canyons, mountainous areas, and plains, and analyzed the physical and socio-historical reasons behind these ratios. Atabeyoğlu [2], based on map fractal analysis, studied the intrinsic correlation between the road network and hydrological network in Turkey and found a high degree of graphical similarity between the two. Tian et al. [3], through buffer zone analysis, compared the different impacts of roads and rivers on the urbanization processes of Guangzhou in China and Phoenix in the United States. Qian et al. [4], using complex network analysis, assessed the reliability of road network connectivity in valley cities and found that the overall connectivity of road networks in these cities is relatively poor and prone to traffic dead ends. Zhang et al. [5], based on geographical information systems, analyzed the relationship between waterfront residential prices in Wuhan and the surrounding road density.

In general, the study of the intrinsic relationship between water bodies and urban transportation has not received extensive attention from the academic community, and a research framework has not yet been established. Current related research mainly involves the impact of roads on river hydrology and ecology, the influence of river floods on urban road networks, and the safety and efficiency of urban cross-river transportation.

Regarding the impact of roads on river hydrology and ecology, researchers primarily focus on road cutting of rivers and the consequences it brings. In this context, Paul Blanton et al. [6] and Wang et al. [7] conducted relevant studies, using examples from the alluvial plains of the United States and the Lancang River Basin in China, respectively. Forman et al. [8] analyzed issues such as hydrological erosion, hydrological disruption, and river sedimentation resulting from road crossings through riverine ecosystems. Roy [9], based on geographic information system data, examined the impact of linear transportation infrastructure on river connectivity in the West Bengal Basin of India and found that 13% of the road network was at risk of flooding during the flood season. Furthermore, Roy et al. [10] also conducted a spatial analysis based on multiple buffer zones to study the impact of road networks on the morphology of river channels in the lowlands of eastern India and found that local river channels experienced adverse effects on hydroecology due to road construction.

Regarding the impact of river floods on urban road networks, some researchers have assessed the harm caused by localized traffic disruptions due to river floods to the normal living order of urban residents. This harm primarily manifests as a reduction in urban road network accessibility and the resulting extension of travel times. For example, Alabbad et al. [11] found that river floods in Iowa, USA, could potentially lead to up to an 18% edge loss in access to major community roads. Rajput et al. [12] discovered that in Harris County, Texas, USA, as little as 1.3% of the road network affected by floods could result in an 8% overall increase in residents’ travel times. In addition, economic losses resulting from river floods have also received attention. Zhou et al. [13] assessed the economic losses caused by river floods to China’s highway network and found that the expected annual losses for Chinese highways in river floods range from $2.04 billion to $3.42 billion. Considering these issues, some researchers have measured the vulnerability of urban road networks to river floods based on the identification of flood-prone areas in cities [14]. This has led to the creation of urban river flood risk maps [15] and proposals for reducing the extent of damage to cities during river floods through the establishment of resilient transportation systems [16].

Regarding the safety and efficiency of urban cross-river transportation, some researchers have calculated the traffic flow of urban river-crossing passages and proposed distribution models for cross-river traffic flow [17, 18] to improve the utilization efficiency of urban river-crossing passages. Moreover, some researchers have proposed economic measures to regulate traffic flow and control congestion in river-crossing passages [19]. Researchers have also focused on the constraints posed by river-crossing passages as vulnerable points in urban road networks on the overall operational efficiency of urban road networks, as well as the impact on urban transportation operations and residents’ travel in the event of accidents or interruptions in river-crossing passages. In this regard, Zhang et al. [20] and Chen et al. [21] found that complex traffic environments and adverse weather conditions significantly affect the safety of driving behavior in river-crossing and underwater tunnels. Scott et al. [22], He et al. [23], and Zhu et al. [24, 25], taking the collapse of urban river-crossing bridges as examples, analyzed the impact of interruptions in cross-river transportation on the travel behavior of urban residents, and found through practical validation that most existing daily traffic distribution models are not suitable for simulating traffic evolution in the event of network interruptions. Outside urban areas, the impact of river-crossing passages on regional transportation and economic structure [26], as well as the sustainability of cross-river transportation in rural areas of developing countries [27] have also received attention from researchers.

Current research indicates that there is a comprehensive and mutually dependent relationship between water bodies and road transportation. The urban road network affects the ecological quality of water bodies [28], while water bodies are related to the overall resilience of the urban road network [29]. However, as mentioned earlier, current research in this field has not focused on how the presence of water bodies affects the evolution of urban road networks, nor has it addressed how water bodies, acting as “barriers”, lead to changes in the layout of urban road networks. Water resources are a prerequisite for urban generation and development, and they may also be a constraint on further urban expansion. The inherent connection between water bodies and cities inevitably has a profound impact on urban road transportation. Therefore, this study focuses on exploring the characteristics and mechanisms of the impact of water bodies on urban road transportation, aiming to fill the current research gap in this relevant field.

## Objects and methods

### Overview of Wuhan’s transportation development under the influence of water

Wuhan, often referred to as the “River Town”, is situated in central China and serves as the capital of Hubei Province. It boasts a unique geographical feature where the Yangtze River, the world’s third-longest river, and the Han River, the largest tributary of the Yangtze, confluence within the city’s core. Furthermore, Wuhan is distinguished by its numerous lakes, earning it the moniker “City of a Hundred Lakes”. The city is extraordinarily abundant in water resources, with a surface water density of 93.456 million m^3^ per square kilometer, significantly surpassing China’s national average of 275,000 m^3^. Additionally, Wuhan’s lakes collectively cover an expansive catchment area of 5,925.2 km^2^, with no less than 65 lakes exceeding 5 km^2^ in catchment area, a distinction that positions it at the forefront among major Chinese cities [30]. Wuhan is also the city with the most vigorous demand for cross-river and cross-lake transportation in China. The first bridge across the Yangtze River was completed and opened to traffic in Wuhan in 1957. As of the end of 2022, Wuhan has constructed or is constructing a total of 13 Yangtze River bridges, with 10 of them spanning the Han River. In addition to these, Wuhan also has completed projects like the East Lake Tunnel and ongoing projects like the Two Lakes Tunnel, both of which are large-scale cross-lake tunnels. Therefore, Wuhan represents an ideal subject for studying the influence of water bodies on urban transportation.

In its formative years, Wuhan comprised three distinct towns named Hankou, Wuchang, and Hanyang, strategically located at the confluence of the Yangtze River and the Han River. Hankou, primarily an economic hub, thrived on waterborne commerce along the Yangtze River. Following its establishment as a port in 1861, Hankou rapidly evolved into an international commercial center, simultaneously serving as China’s financial and logistical nucleus. During this era, Hankou’s adjacency to both the Yangtze River and the Han River facilitated shipping operations. The town assumed a linear configuration, with key thoroughfares like Poyang Street and Dongting Street running parallel to the rivers, while cross-streets converged upon the riverside wharves. This network of intersecting streets delineated Hankou into numerous compact square communities, colloquially referred to as “Lifen”. Wuchang, situated in close proximity to the Yangtze River and serving as the capital of Hubei Province, found itself surrounded by an array of expansive lakes, including Sha Lake, East Lake, South Lake, Jia Lake, and Qingling Lake on its north, east, and south flanks. These abundant water bodies imposed geographical constraints on Wuchang’s transportation networks, resulting in radial road systems. For instance, main arteries extended outward from the town between Sha Lake and the Yangtze River, East Lake and South Lake, South Lake and Jia Lake, and Jia Lake and the Yangtze River, all converging within the boundaries of Wuchang City. Hanyang, similarly situated alongside both the Han River and the Yangtze River, featured a road network aligned with these two major watercourses. The town’s outer perimeter was encircled by Moon Lake, Longyang Lake, Moshui Lake, Tudi Lake, and Taizi Lake, resulting in a comparatively smaller expanse of developable land compared to Hankou and Wuchang.

In 1927, a significant milestone was reached when the formerly independent municipalities of Hankou, Wuchang, and Hanyang merged, forming the nucleus of what we now know as Wuhan. This merger marked the initiation of a stage characterized by integrated and coordinated development among the three towns. In 1957, a pivotal development unfolded with the inauguration of the Wuhan Yangtze River Bridge, which formally amalgamated the road networks of these three towns. Subsequently, Wuhan embarked on a large-scale land development and industrialization drive, leading to the gradual outward expansion of the city. This expansion posed a formidable challenge, as the numerous surrounding lakes inevitably became obstacles to the city’s continued growth. During the 1950s to 1970s, extensive reclamation efforts resulted in a reduction of lake area by 154.6 km^2^ in Wuhan, accounting for 60% of the total lake area at that time. The 1980s witnessed the commencement of a substantial lake-filling and urban development initiative in Wuhan. This endeavor further reduced the city’s water bodies, with the number of lakes plummeting from 127 in 1950 to 40 by 2010. The total lake area diminished by 228.9 km^2^. Additionally, this lake-filling and urban development endeavor severed many natural connections between the lakes, particularly those linking to the Yangtze River and the Han River. Furthermore, it exacerbated water pollution issues. As the city expanded, lakes once on its periphery, such as Sha Lake, East Lake, and South Lake, were engulfed by urban sprawl, transforming them into integral downtown features. Simultaneously, the impeding effect of lakes on urban transportation became a pressing concern, affecting the efficiency of the urban road network. In recent years, Wuhan has deployed three primary strategies to alleviate cross-lake transportation challenges: lakeside roads, elevated viaducts, and sub-lake tunnels. These measures have mitigated urban traffic congestion to some extent.

### Research source

We have chosen four city maps representing distinct developmental stages in the past century of Wuhan’s evolution as the focal points for our analysis. Wuhan has a rich urban development history. From the 1920s to the present, the city has undergone construction in multiple periods. The older urban areas, especially in the Hankou region, have preserved numerous historical streets, alleys, and buildings. Similarly, the towns of Wuchang and Hanyang also boast the retention of historical architecture. We use OpenStreetMap (https://www.openstreetmap.org/) road network data and water body data as a foundation. By utilizing the existing historical buildings and streets in Wuhan as spatial reference points, we can compare historical maps with current map information. Combining maps of Wuhan from four different periods, we utilized ArcGIS software (ESRI Corporation, Redlands, CA) to create vector representations of the road network and water bodies for each of the four periods. The four selected maps are as follows:

The 1922 edition of the “Wuhan Three Towns Street Map”, which currently resides in the Beijing Library, included in Wuhan Historical Atlas [31]. This map from the Republic of China era reflects a period of robust economic activity within Wuhan’s three towns, profoundly reliant on waterborne transportation via the Yangtze River and the Han River.

The 1969 edition of the “Wuhan Street Map”, which can be retrieved from Wikimedia Commons [32]. During this era, Wuhan underwent extensive industrial development and land expansion following the establishment of the People’s Republic of China. The city’s scale experienced remarkable enlargement compared to pre-PRC times, while the nexus between transportation and rivers entered a phase of diminishing significance.

The 1995 edition of the “Satellite Impact Map of Wuhan Metropolitan Area”, also available in the Wuhan Survey and Design Institute, included in Wuhan Historical Atlas [31]. In this period, Wuhan underwent further expansion following the onset of economic reforms and opening up. Simultaneously, the city witnessed a series of initiatives involving lake infill and urban development. This led to a substantial reduction in the city’s water areas compared to the early years of the PRC, with water bodies exerting a constraining influence on urban transportation development.

The 2023 edition of the “Wuhan City Map”, sourced from the Hubei Provincial Platform for Common GeoSpatial Information Services [33]. In the current era, Wuhan is vigorously pursuing cross-river and cross-lake transportation initiatives. Concurrently, there is an unprecedented emphasis on the preservation of lakes and their surrounding ecological environments. The development of greenways encircling these lakes is actively encouraging citizens to engage more closely with urban water bodies. Consequently, Wuhan presently finds itself in a stage characterized by the pursuit of mutually beneficial development between urban transportation and water resources.

### Establishment of water buffer zones and associated analysis

To examine the evolving relationship between water bodies and urban transportation in Wuhan over the past century, we employed ArcMap to create vectorized datasets for the aforementioned four distinct time periods, encompassing water bodies and urban road networks. Typically, roads located within approximately 1 kilometer of rivers demonstrate a close correlation with the adjacent water bodies, while roads within a proximity of approximately 500 meters to lakes exhibit a strong association with these lakes. Consequently, utilizing ArcMap, we established a 1-kilometer buffer zone along the Yangtze River and the Han River. Recognizing that lakes with smaller water areas tend to exert negligible practical influence on urban transportation dynamics, we opted to apply a 500-meter buffer zone exclusively to 15 urban lakes with water areas exceeding 1 square kilometer. Subsequently, we quantified the proportion of road length contained within these buffer zones relative to the total road length across the city during the four specified temporal intervals. This analysis enabled us to discern the changing trends in this proportion. The formula is as follows:

$$\mu =l_{b}/l_{t}$$ (1)

In the formula, *l*_*b*_ represents the length of roads within the buffer zone, and *l*_*t*_ signifies the total road length within urban areas. Consequently, a higher value of *μ* denotes a more pronounced correlation between urban road networks and water bodies.

### Urban regional accessibility analysis utilizing road distance/spatial distance matrices

Hansen introduced the concept of “accessibility” [34], which pertains to the ease of travel between fixed points within a given transportation system. Presently, widely adopted accessibility evaluation models include distance models, gravity models, and opportunity models. While quantitative assessments of road network accessibility have gained prominence, much of the research has predominantly focused on macro or meso-level analyses. In international [35], national [36–38], or regional [39–41] urban conglomerations, researchers often employ network topology to evaluate various accessibility aspects between cities, treating these cities as nodes. However, micro-level investigations, such as inter-regional accessibility within a single city, remain relatively scarce. Furthermore, establishing rational nodes for evaluating accessibility within specific city areas poses a considerable challenge.

Considering the speed differences among different modes of transportation, time-distance models are preferred over the shortest path models for macro or meso-level accessibility assessments conducted with urban areas as nodes. For instance, Chen et al. [39] assessed accessibility in the Beijing-Tianjin-Hebei region’s overland road network, and Liu et al. [36] evaluated spatial accessibility in the Wuhan metropolitan area’s road network. In the assessment of road accessibility within the city, some researchers have considered the differences in transportation efficiency among different road grades and modes of transport, as well as the impact of urban land use. Gravity models and opportunity models have been introduced based on these considerations. For instance, Duccio et al. [42] found a significant attenuation gap in BRT routes compared to other routes in urban bus rapid transit systems. Sarah et al. [43], through a comparative analysis of accessibility for various modes of travel, found that the gravity model is significantly applicable to situations with different path efficiencies. Zhai et al. [44], based on gravity models and opportunity models, conducted a comparative analysis of accessibility before and after the construction of urban subways, where traffic speed and transportation efficiency were identified as important influencing factors. This study focuses on the impact of water bodies on the evolution of urban road network structure and the inherent relationship between the corresponding road network structure and the overall accessibility of urban roads. The urban road network structure also affects residents’ travel efficiency [45], travel safety [46], and travel energy consumption [47]. The shortest path model is more suitable than other accessibility assessment models for illustrating the direct impact of urban road network structure on road accessibility. Therefore, this study adopts the shortest path model based on its focus on the urban road network structure itself. Traditional shortest path models, like the shortest distance matrices constructed by Jin et al. [48, 49] and Wang et al. [38], are commonly employed:

$$M={\left[d_{ij}\right]}_{n\times n}$$ (2)

In the formula, *d*_*ij*_ signifies the shortest road distance, measured in kilometers (km), from location node *i* to location node *j* within a city, considering a finite number of such nodes. This road distance serves as the fundamental unit for the matrix.

By aggregating calculations across all nodes, we ultimately construct a connectivity matrix depicting the shortest paths between every pair of nodes. Consequently, we can define the total road distance of node *i* as follows:

$$D_{i}=\sum d_{ij}(j=1,2,3,\cdots ,n)$$ (3)

In the formula, *D*_*i*_ represents the cumulative shortest-path distance from a specific node within the city to all other designated nodes. A lower value signifies enhanced accessibility of the node within the city. The measure of accessibility strength for a node is denoted as the accessibility coefficient *A*_*di*_. It is calculated as the ratio of the total road distance (*D*_*i*_) for a particular node within the city to the average total road distance (*D*_*i*_) across all city nodes. The formula is as follows:

$$A_{di}=D_{i}/\left(\sum D_{i}/n\right)\;\left(i=1,2,3,\cdots ,n\right)$$ (4)

In the formula, *A*_*di*_ serves as an indicator of the relative accessibility of various nodes within the entire city. Smaller values correspond to improved node accessibility. If *A*_*di*_ exceeds 1, it signifies that the node’s accessibility is below the city-wide average, while values below 1 indicate accessibility exceeding the city’s average.

Nonetheless, calculations based on the shortest path model primarily reflect the locational advantages and disadvantages of nodes. This limitation hinders the complete expression of environmental characteristics impacting accessibility. Consequently, assessing the impact of water bodies, acting as “obstacles”, on the urban road network using the traditional shortest path model becomes challenging.

Therefore, we introduce a novel approach, utilizing road distance/spatial distance matrices, to assess urban road accessibility based on their inherent environmental conditions. In contrast to the traditional shortest path model, this approach effectively mitigates location-related biases among city nodes, which significantly influences accessibility evaluation. This methodology allows for a more intuitive analysis of how rivers and lakes might influence urban road accessibility. For the road distance/spatial distance matrix *L*:

$$L={\left[d_{ij}/s_{ij}\right]}_{n\times n}$$ (5)

In the formula, *d*_*ij*_ signifies the shortest road distance in kilometers (km) from location node *i* to location node *j* within the city. Conversely, *s*_*ij*_ denotes the Euclidean distance in kilometers (km) between these two points, without accounting for traffic conditions. Within this formula, when *d*_*ij*_⁄*s*_*ij*_ ≥ 1, a higher value indicates a more convoluted route from point *i* to point *j*. By aggregating calculations across all nodes within the city, a connect matrix representing the road distance/spatial distance between every pair of nodes can be generated. Furthermore, the average road distance/spatial distance between node *i* and all other city nodes can be defined as follows:

$$\bar{L_{i}}=\frac{\sum \left(d_{ij}/s_{ij}\right)}{n}(j=1,2,3,\cdots ,n)$$ (6)

The term $\bar{L_{i}}$ can be aptly referred to as the “Detour Index” for node *i* within the city. As per formula 6, it’s evident that $\bar{L_{i}}\geq 1$. A higher $\bar{L_{i}}$ value indicates reduced accessibility for node *i* based on its inherent environmental conditions. This also implies a greater likelihood of single or multiple unfavorable factors impacting the node’s accessibility in the region, necessitating longer detours to reach other areas.

To facilitate a more precise comparison of node accessibility within the city, considering their individual environmental characteristics, we introduce the “Weighted Detour Index” denoted as $A_{\bar{li}}$ for node *i*. This index is calculated by dividing the $\bar{L_{i}}$ value of node *i* by the average $\bar{L_{i}}$ value of all nodes in the city. The formula is as follows:

$$A_{\bar{li}}=\frac{\bar{L_{i}}}{\sum \bar{L_{i}}/n}\;\left(i=1,2,3,\cdots ,n\right)$$ (7)

In the formula, $\sum \bar{L_{i}}/n$ represents the average $\bar{L_{i}}$ value for all nodes within the city. When $A_{\bar{li}}$ is greater than 1, it signifies that node *i*’s accessibility based on its inherent environmental conditions falls below the average accessibility level of all city nodes. Conversely, when $A_{\bar{li}}$ is less than 1, it indicates that node *i*’s accessibility based on its inherent environmental conditions surpasses the average accessibility level of all city nodes.

Utilizing the described model and the previously mentioned cartographic resources, namely the 1969 edition of the “Wuhan Street Map”, the 1995 edition of the “Satellite Impact Map of Wuhan Metropolitan Area”, and the 2023 edition of the “Wuhan City Map”, the central urban areas depicted in these three vectorized maps were subdivided into a grid measuring 1km×1km. Typically, within each 1 km^2^ grid, the accessibility of individual nodes does not exhibit significant disparities. Therefore, we selected one representative transportation node for each grid, excluding those situated in waterlogged, mountainous, or non-accessible regions.

Over the past century, Wuhan’s urban footprint has continuously expanded, leading to dynamic changes in the central city area’s size. Consequently, we identified 97 nodes in the 1969 map, 197 nodes in the 1995 map, and 445 nodes in the 2023 map. Subsequently, we calculated the $\bar{L_{i}}$ and $A_{\bar{li}}$ values for all nodes within these three maps. The resulting data were aggregated and analyzed to elucidate the potential impact of rivers and lakes on urban road accessibility throughout various stages of urban transportation development. Notably, our analysis excludes the 1922 version of the “Wuhan Three Towns Street Map” due to the absence of Yangtze River and Han River bridges at that time, along with the independent transportation systems of the three towns.

## Results and discussion

### Dynamics of riverside and lakeside transportation during urban expansion

**Fig 1:**
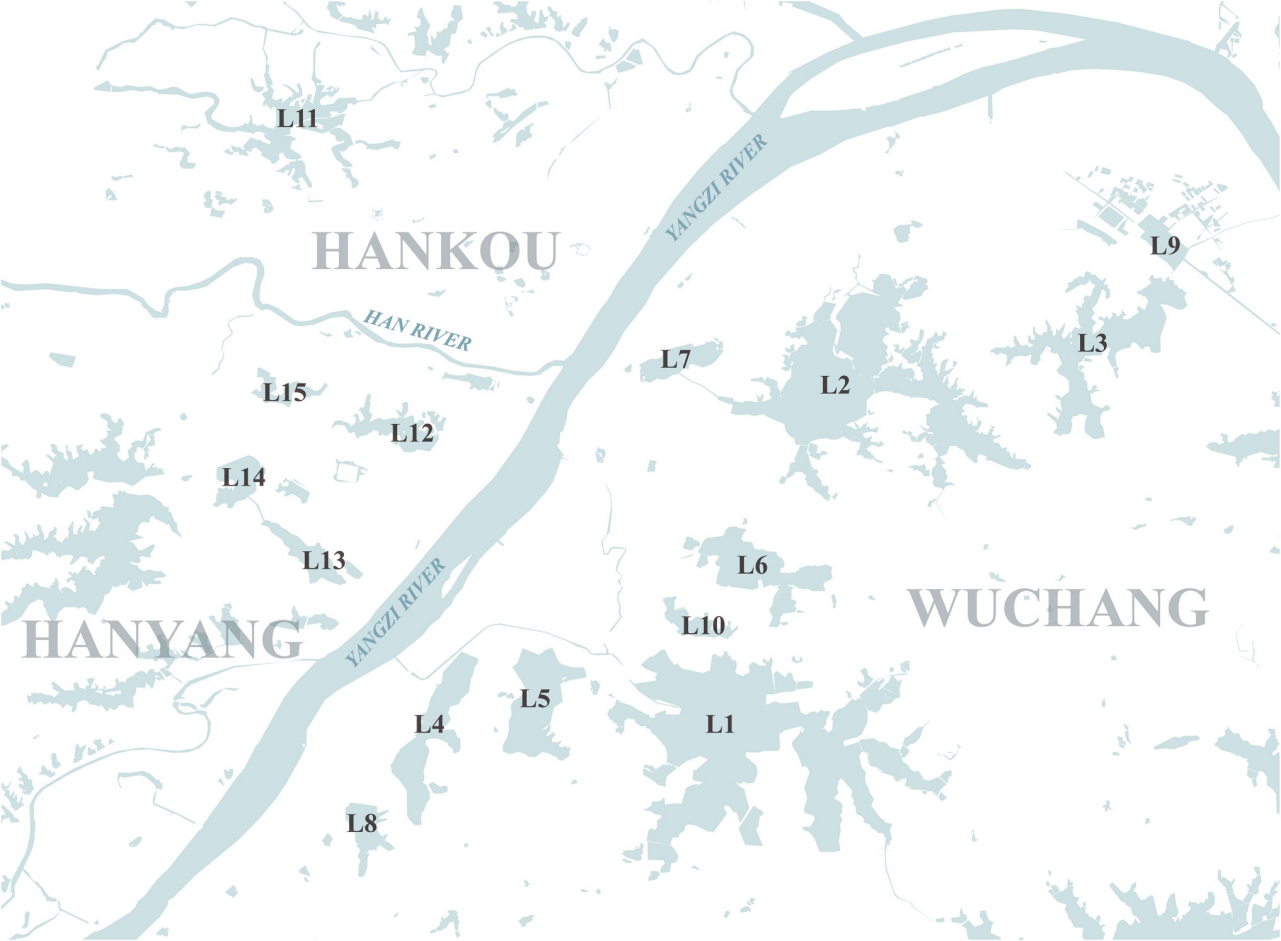
The urban water bodies in Wuhan analyzed in this study. (L1) Tangxun Lake; (L2) East Lake; (L3) Yanxi Lake; (L4) Qingling Lake; (L5) Huangjia Lake; (L6) South Lake; (L7) Sha Lake; (L8) Ye Lake; (L9) Qingshanbei Lake; (L10) Yezhi Lake; (L11) Jinyin Lake; (L12) Moshui Lake; (L13) Nantaizi Lake; (L14) Sanjiao Lake; (L15) Longyang Lake. Base map and data from OpenStreetMap and OpenStreetMap Foundation.

Fig 1 represents the urban water bodies in Wuhan analyzed in this study. The primary water bodies influencing transportation within Wuhan’s urban area are the Yangtze River and its major tributary, the Han River. To significantly impact the city’s road network, lakes must possess substantial surface areas. Consequently, our analysis focused solely on 15 lakes within the urban area of Wuhan, each with a current water surface exceeding 1 square kilometer, for the investigation of lakeside transportation.

#### Riverside transportation

**Fig 2:**
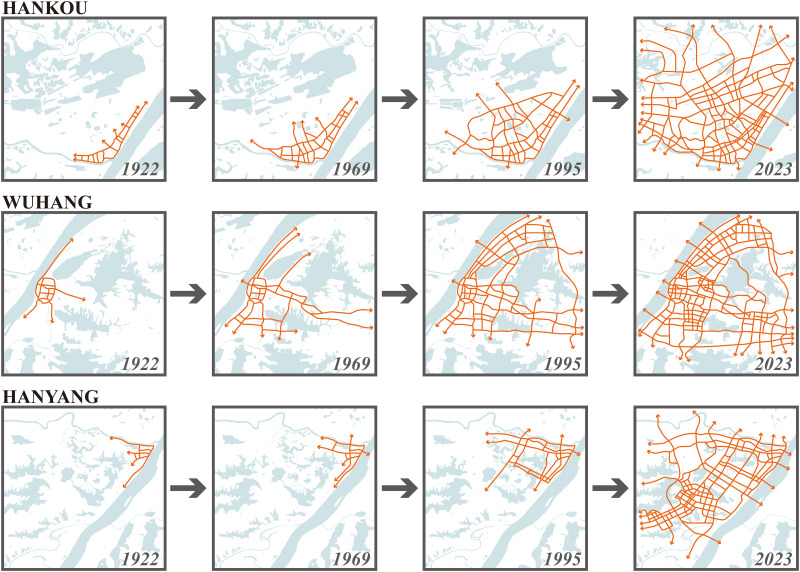
Evolution of urban road network in Hankou, Wuchang and Hanyang. Base map and data from OpenStreetMap and OpenStreetMap Foundation.

**Fig 3:**
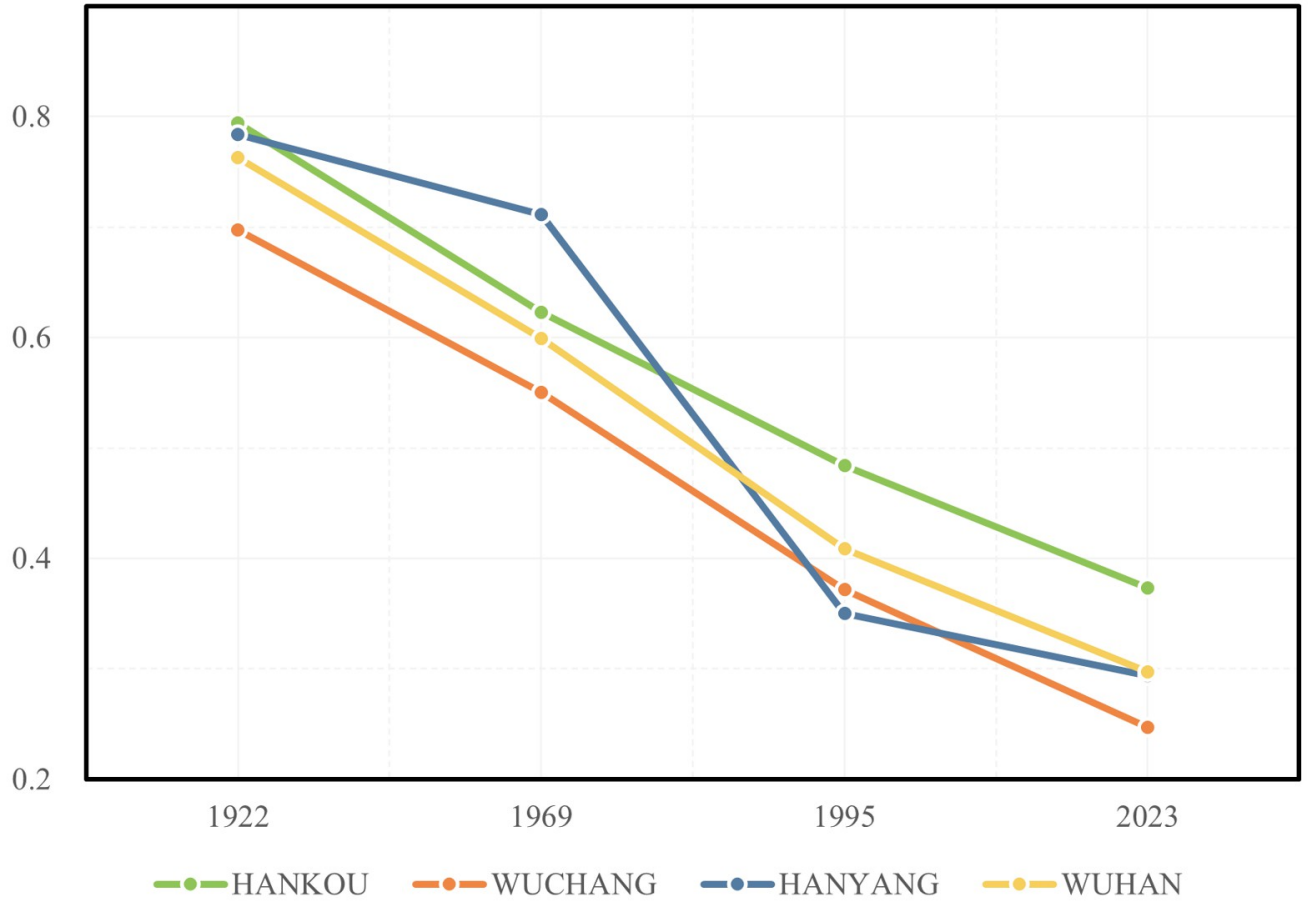
Trends in the proportion of road lengths within the river buffer zone in relation to the total length of urban roads within Wuhan.

Table 1 and Figs 2 and 3 illustrate a consistent decline in the proportion of riverside road lengths relative to the total urban road network in Wuhan’s three towns from 1922 to 2023. In 1922, Hankou and Hanyang were intricately tied to the waterways of the Yangtze River and Han River. Hankou primarily thrived as a commerce hub, while Hanyang focused on industrialization. Consequently, the lengths of riverside roads in Hankou and Hanyang accounted for 79.4% and 78.4%, respectively, whereas Wuchang’s share stood at 69.7%. However, the subsequent expansion of Wuhan’s urban area did not rigidly follow the riverside. Instead, it extended inland from the three towns, resulting in a less linear urban layout. As development shifted away from the riverbanks, in 2023, the length of riverside roads in Wuhan accounted for only 29.7% of the total road length in the city, with Wuchang accounting for just 24.7%. The urban development of Hankou was based on its business layout adjacent to the Yangtze River and Han River. The original main roads were built along the river, as the city expanded, new roads were constructed parallel to Yanjiang Avenue and gradually shrank towards the urban hinterland and were perpendicular to the various main roads across the river later, forming the urban road network of Hankou together. Wuchang’s development radiated outward from the older Yangtze River area. However, the presence of numerous large lakes in Wuchang resulted in a more dispersed urban layout compared to Hankou. This dispersion gave rise to a multi-center cluster development pattern interconnected by long-distance main roads, forming Wuchang’s transportation skeleton. In contrast to Hankou’s riverside-centric approach, Wuchang’s transportation layout did not rely heavily on riverside roads from its inception, contributing to a consistently lower proportion of riverside road lengths relative to the total road network. Hanyang’s initial urban layout also bordered the Yangtze River and Han River, but its riverside roads played a less prominent role due to the absence of substantial riverside commerce. Instead, Hanyang adopted a square-shaped urban road network in its hinterland, facilitating modern industrial development. Hanyang’s urban expansion was relatively slower than Hankou and Wuchang, with significant hinterland development commencing in the 1990s. Consequently, the proportion of riverside road lengths relative to the total road network in Hanyang significantly decreased from 71.1% in 1969 to 35.0% in 1995.

**Table 1:** Statistics of urban road network changes within the river buffer zone in Wuhan.

**Years**	**Hankou**				**Wuchang**			
	Buffer area (km^2^)	Urban road length (m)	Buffer road length (m)	Buffer road length / Total length of urban roads	Buffer area (km^2^)	Urban road length (m)	Buffer road length (m)	Buffer road length / Total length of urban roads
**1922**	10.5	155.6	123.5	0.794	8.2	80.6	56.2	0.697
**1969**	51.7	392.2	244.3	0.623	44.6	427.1	234.9	0.550
**1995**	52.2	580.5	280.9	0.484	49.0	820.1	305.4	0.372
**2023**	60.0	986.2	358.2	0.373	66.7	1,528.2	377.9	0.247
**Years**	**Hanyang**				**Whole Wuhan**			
	Buffer area (km^2^)	Urban road length (m)	Buffer road length (m)	Buffer road length / Total length of urban roads	Buffer area (km^2^)	Urban road length (m)	Buffer road length (m)	Buffer road length / Total length of urban roads
**1922**	4.2	28.2	22.1	0.784	22.9	264.4	201.8	0.763
**1969**	29.0	103.4	73.5	0.711	125.3	922.7	552.7	0.599
**1995**	33.9	225.4	78.9	0.350	135.1	1,626.0	665.2	0.409
**2023**	46.1	437.4	139.3	0.294	172.8	2,951.8	875.4	0.297

#### Lakeside transportation

**Fig 4:**
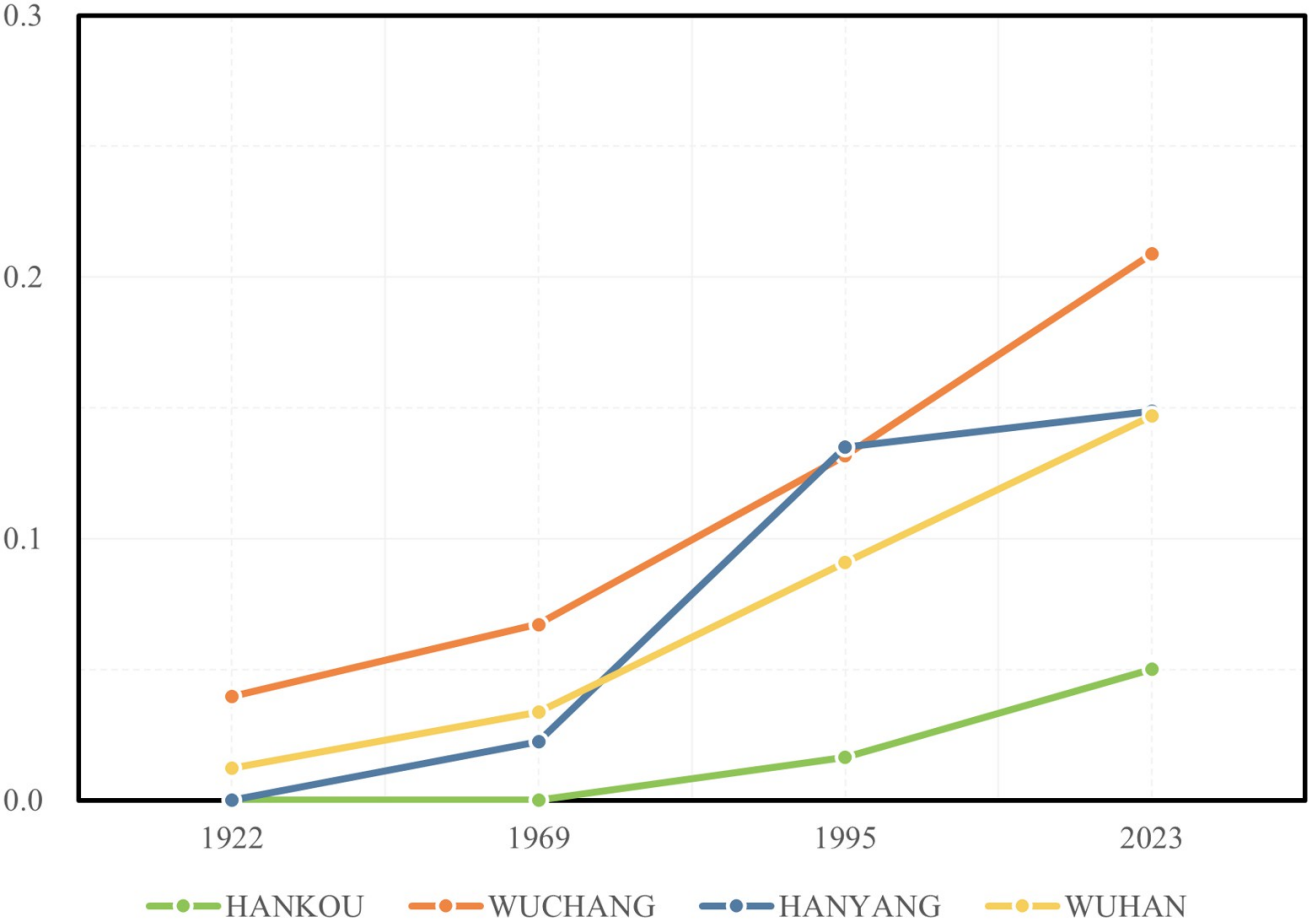
Trends in the proportion of road lengths within the lake buffer zone in relation to the total length of urban roads within Wuhan.

Initially, there was no discernible correlation between the city’s layout and the presence of larger lakes during Wuhan’s early development. Table 2 and Fig 4 illustrate the proportion of lakeside road lengths relative to the total urban road length in 1922, which was a mere 1.2%. At that time, the larger lakes were predominantly situated on the city’s outskirts, hindering further expansion. However, by 1969, lakes such as Moshui Lake, Sha Lake, East Lake, and South Lake, located closer to the city center, began encroaching upon the expanding urban area. Notably, Moshui Lake in Hanyang obstructed southwestern expansion, while Sha Lake, East Lake, and South Lake in Wuchang impeded northeastern and southeastern growth. Consequently, new urban development in Wuhan was primarily organized linearly between bodies of water. Numerous long-distance main roads extended outward from the city center into the emerging urban area, such as Changzheng Avenue (now known as Hanyang Avenue), Hanyang Avenue (now Yingwu Avenue), Renmin Avenue (now Linjiang Avenue), Dongfeng Avenue (now Heping Avenue), Hongqi Avenue (now Wuluo Road and Luoyu Road), etc. However, these main roads were often disconnected due to the presence of water bodies. Therefore, even though the city had expanded, it was unable to establish an efficient road network. During this phase, lakeside transportation had not been fully integrated into the urban road network planning, consistently bypassing lakes. As a result, the proportion of lakeside road length relative to the total road length of the city only saw a modest increase, reaching 3.4%. However, as Wuhan continued to expand further, some of the larger lakes became encompassed within the city limits. Concurrently, additional lakes in the outer suburbs began to play a role in urban transportation, leading to a significant surge in the number of lakeside roads. By 1995, the proportion of lakeside road length to the total urban road length had risen substantially to 9.1%. In 2023, this proportion had further increased, reaching 14.7% within the total length of urban roads in Wuhan.

**Table 2:** Statistics of road changes in the lake buffer zone in Wuhan.

	Numbering	Lake name	Water area *S* in 2023 (km^2^)	Road length *l* with the buffer (km)				Lakeside road density *d* in 2023 (*d* = *S*/l_2023_)
				1922	1969	1995	2023	
**Wuchang**	**L1**	**Tangxun Lake**	47.62	/	/	22.4	123.39	2.59
	**L2**	**East Lake**	32.80	/	17.6	43.9	71.94	2.19
	**L3**	**Yanxi Lake**	14.23	/	/	8.8	35.07	2.46
	**L4**	**Qingling Lake**	8.84	/	/	3.1	11.92	1.35
	**L5**	**Huangjia Lake**	8.12	/	/	2.9	10.21	1.26
	**L6**	**South Lake**	7.67	/	5.4	12.5	26.04	3.40
	**L7**	**Sha Lake**	3.08	3.2	6.5	7.8	18.26	5.93
	**L8**	**Ye Lake**	3.00	/	/	/	6.37	2.12
	**L9**	**Qingshanbei Lake**	1.94	/	/	/	4.81	2.48
	**L10**	**Yezhi Lake**	1.62	/	/	6.5	11.07	6.83
		**Total**	128.92	3.2	28.7	107.9	319.08	/
**Hankou**	**L11**	**Jinyin Lake**	8.16	/	/	9.6	49.35	6.05
		**Total**	8.16	/	/	9.6	49.35	/
**Hanyang**	**L12**	**Moshui Lake**	3.64	/	2.3	15.6	27.85	7.65
	**L13**	**Nantaizi Lake**	3.57	/	/	3.3	13.66	3.83
	**L14**	**Sanjiao Lake**	2.39	/	/	7.2	11.39	4.77
	**L15**	**Longyang Lake**	1.68	/	/	4.3	12.17	7.24
		**Total**	11.28	/	2.3	30.4	65.07	/
		**Whole total**	148.36	3.2	31.0	147.9	433.50	/

Integrating lakes, as valuable water resources, into the city is crucial, and overcoming the historic “constraint” imposed by lakes on urban transportation is a prerequisite for effective urban expansion. In the past, numerous lakes in Wuhan were filled to expand land for urban development, resulting in a significant reduction in the city’s water area. In recent years, transportation across these lakes has primarily been addressed using three methods: roads encircling the lake, viaducts, and tunnels beneath the lake. However, the dense network of roads near lakes can adversely impact the lake’s ecosystem and biodiversity. In response to this, Wuhan implemented the “Three Lines and One Road” plan to safeguard the ecological buffer zone between urban roads and lakes. Table 2 illustrates that lakeside road density is generally higher in the city’s central area. For example, within Wuhan’s Second Ring Road, the road density around Sha Lake in 2023 is 5.93 km/km^2^; Moshui Lake, Yezhi Lake, and South Lake, located between the Second Ring Road and Third Ring Road, exhibit densities of 7.65 km/km^2^, 6.83 km/km^2^, and 3.40 km/km^2^, respectively. Conversely, the road density near East Lake is only 2.19 km/km^2^ due to its expansive water area. In the outer suburbs of Wuhan, lakeside road density is generally lower, as seen around Tangxun Lake, Qingling Lake, Huangjia Lake, Ye Lake, Qingshanbei Lake, Yanxi Lake, and others. This is because the level of development around these lakes still lags behind the urban areas. However, the road density around Jinyin Lake in the northwestern suburbs of Hankou is notably high at 6.05 km/km^2^ because it is the only lake with a well-developed road system among the 15 larger lakes in Wuhan city. These roads encircling the lakes serve as the core of the transportation network, connecting the surrounding areas and effectively promoting industrial development along the lakeshores. Consequently, the degree of land development around Jinyin Lake surpasses those around most other outer suburban lakes in Wuhan.

#### Overall trends in waterfront transportation

**Fig 5:**
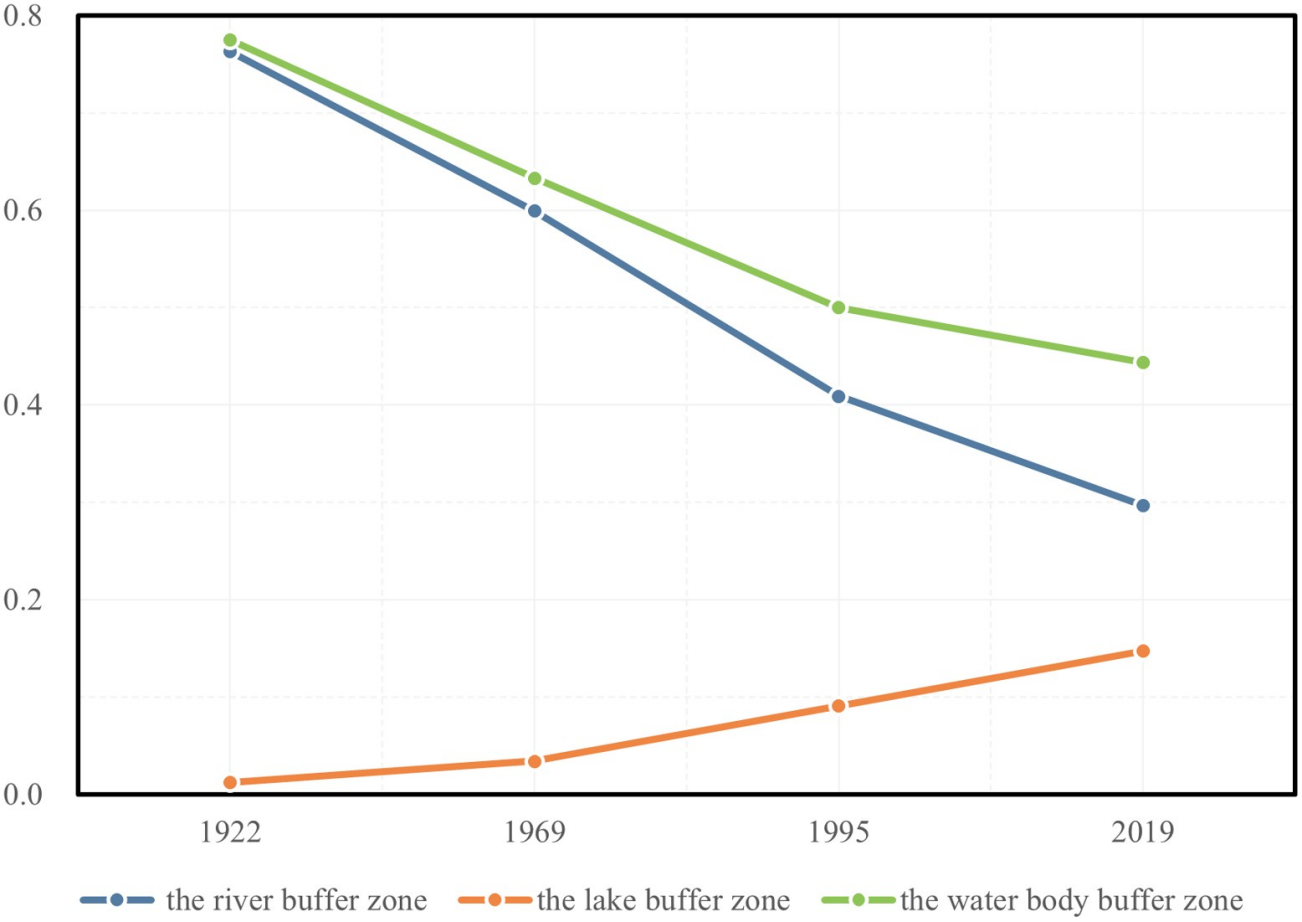
Trends in the proportion of road lengths within the lake buffer zone in relation to the total length of urban roads within Wuhan.

As shown in Fig 5, over the past century, during the continuous urban expansion of Wuhan, the correlation between urban water bodies and urban transportation has been consistently weakening. Within the riverside buffer zone, the proportion of road length to the total urban road length has gradually decreased, declining from 76.3% in 1922 to 29.7% in 2023. However, the proportion of road length within the lakeside buffer zone has increased from 1.2% in 1922 to 14.7% in 2023. Meanwhile, the proportion of road length within the entire urban water body buffer zone has decreased from 77.5% in 1922 to 44.3% in 2023. It can be inferred that with the urban expansion of Wuhan, the traditional urban road network relying on water transportation has disintegrated, and rivers are no longer the core factor influencing the development of urban transportation. However, the lakes that were originally located outside the city have formed a more direct connection with the urban road network, which may also become an obstacle to further expansion of the urban road network. Therefore, coordinating the relationship between urban water bodies and urban transportation remains crucial in the current context.

### Evolutionary characteristics of urban regional accessibility when water bodies act as “obstacles” in Wuhan

#### The influence process of water distribution on urban transportation development

The presence of water bodies has always profoundly influenced the development of transportation in Wuhan. This influence is most evident in the continuous expansion of the city’s urban road network, which had to overcome the formidable “obstacle” posed by water bodies. Back in 1922, Wuhan was divided into three distinct towns: Hankou, Wuchang, and Hanyang, each separated by rivers. During this era, the sole mode of transportation connecting these towns was via ferries. This water barrier effectively isolated the three towns from each other, leading to political and economic independence. Furthermore, the large lakes surrounding Wuhan were not integrated into the urban transportation system at that time, so the road networks within the three towns were well-developed and remained unaffected by water bodies. The pivotal Jianghan Bridge, connecting Hankou and Hanyang, was completed in 1956, followed by the Wuhan Yangtze River Bridge linking Hanyang and Wuchang in 1957. Consequently, by 1969, the road networks of the three towns were seamlessly integrated, culminating in the transformation of Wuhan into a unified city formed by the merger of these towns. During this transition, the role of water bodies acting as the “obstacle” to urban transportation development was weakened for the first time, thanks to the Yangtze River and Han River bridges that ensured vital connections. However, travel between the northern parts of Hankou and Wuchang still necessitated considerable detours, with cross-river ferries remaining essential. On another front, the lakes began to impede urban expansion, as the separation of rivers and lakes prevented direct road connections between some new urban areas. The year 1995 marked a turning point with the successive completion of the Second Jianghan Bridge (now known as the Zhiyin Bridge) and the Second Wuhan Yangtze River Bridge. These developments connected road network in Wuhan into a formal ring, coinciding with the incorporation of several large lakes within the city. During this period, the role of water bodies as an “obstacle” to urban transportation development continued to be weakened, but at the same time, the impact of water bodies on the urban transportation development becomes more diverse. As of 2023, Wuhan boasts 11 bridges and 2 road tunnels spanning the Yangtze River, along with 9 bridges across the Han River, forming an integrated road network throughout the city. Furthermore, roads across the lakes have been seamlessly integrated into the urban road network. Today, water bodies are no longer the primary obstacle to urban transportation expansion. However, its enduring influence on regional accessibility remains a significant factor that profoundly impacts future urban road network optimization and industrial layout.

#### The evolution characteristics of urban regional accessibility under the influence of the water bodies

**Fig 6:**
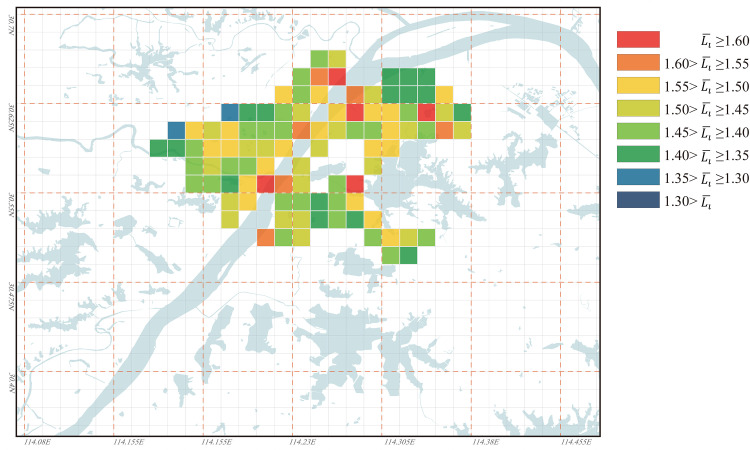
$\bar{L_{l}}$ values distribution for all nodes in Wuhan in 1969. Base map and data from OpenStreetMap and OpenStreetMap Foundation. (Data presented in S1 Table in S1 File).

**Fig 7:**
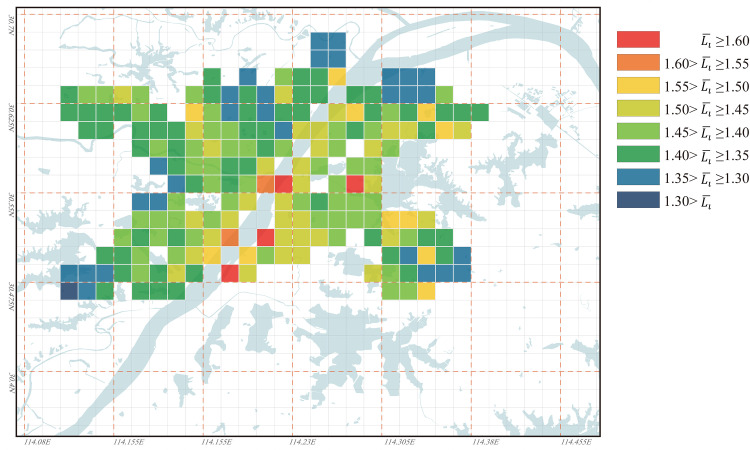
$\bar{L_{l}}$ values distribution for all nodes in Wuhan in 1995. Base map and data from OpenStreetMap and OpenStreetMap Foundation. (Data presented in S2 Table in S1 File).

**Fig 8:**
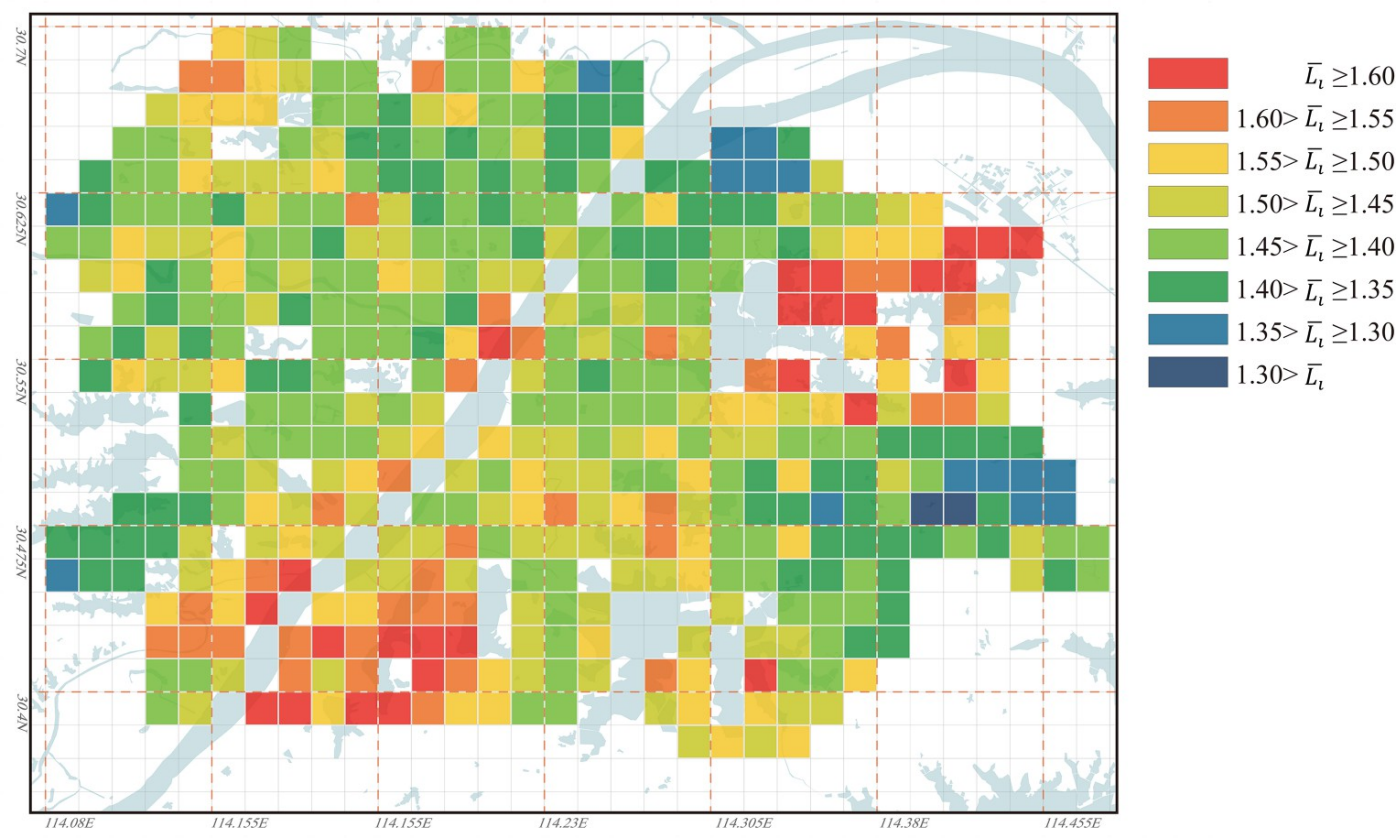
$\bar{L_{l}}$ values distribution for all nodes in Wuhan in 2023. Base map and data from OpenStreetMap and OpenStreetMap Foundation. (Data presented in S3 Table in S1 File).

The distribution of the $\bar{L_{i}}$ values of all nodes in Wuhan for the years 1969, 1995, and 2023 is illustrated in Figs 6–8. From the figures, it can be observed that in 1969, Wuhan’s cross-river transportation system was not well-established. The city had only one passage across the Yangtze River and one passage across the Han River, resulting in a generally high $\bar{L_{i}}$ value. Most nodes had $\bar{L_{i}}$ values above 1.40, and the distribution of $\bar{L_{i}}$ values in the city was unbalanced. In 1995, as Wuhan’s cross-river transportation network became more complete, the overall $\bar{L_{i}}$ value significantly decreased, and its distribution became more balanced. Some nodes had $\bar{L_{i}}$ values below 1.40, with only a few areas along the banks of Yangtze River having higher $\bar{L_{i}}$ values. By 2023, although Wuhan’s cross-river transportation system had further improved compared to 1995, some areas within the city had $\bar{L_{i}}$ values exceeding 1.60. This is mainly due to the extensive urban expansion, where lakes that were originally outside the city became part of the urban area but were impacted by the presence of water bodies, resulting in poor accessibility. This unbalanced distribution of $\bar{L_{i}}$ values in the city was more pronounced than in 1995.

**Fig 9:**
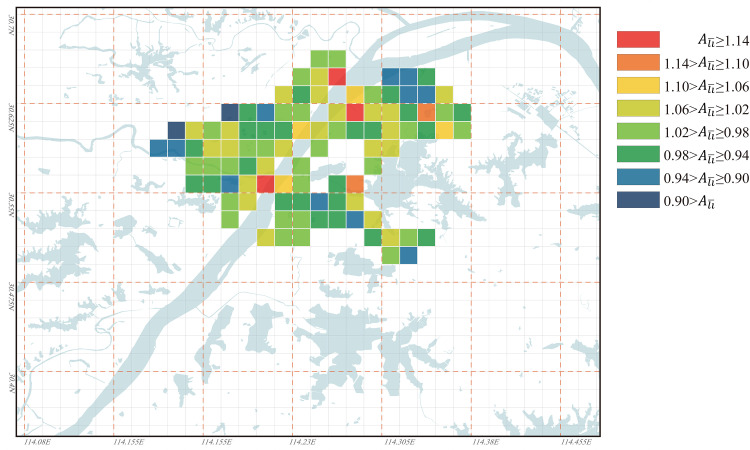
$A_{\bar{li}}$ values distribution for all nodes in Wuhan in 1969. Base map and data from OpenStreetMap and OpenStreetMap Foundation. (Data presented in S1 Table in S1 File).

**Fig 10:**
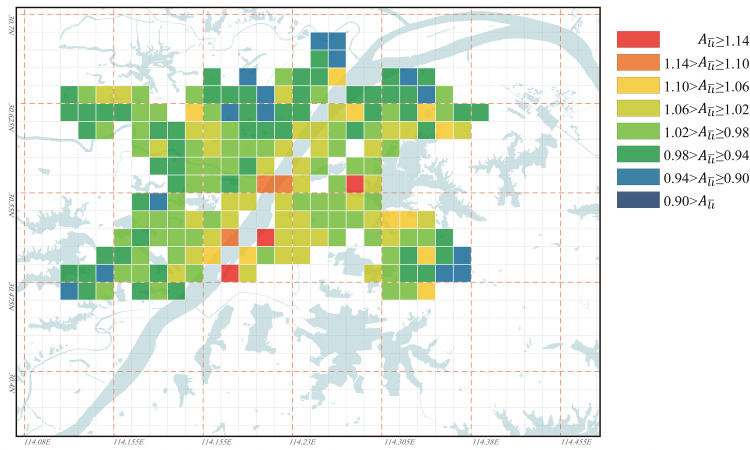
$A_{\bar{li}}$ values distribution for all nodes in Wuhan in 1995. Base map and data from OpenStreetMap and OpenStreetMap Foundation. (Data presented in S2 Table in S1 File).

**Fig 11:**
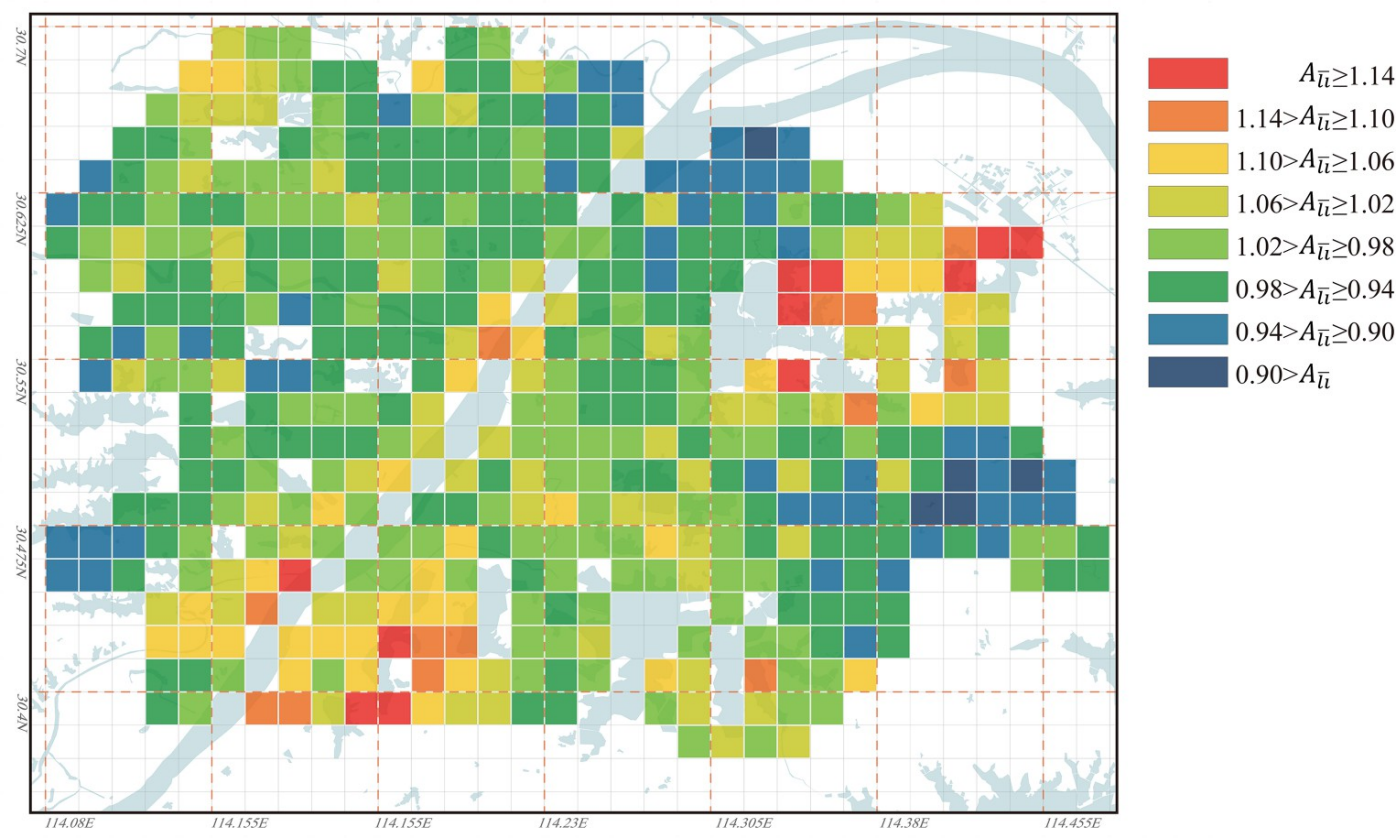
$A_{\bar{li}}$ values distribution for all nodes in Wuhan in 2023. Base map and data from OpenStreetMap and OpenStreetMap Foundation. (Data presented in S3 Table in S1 File).

**Fig 12:**
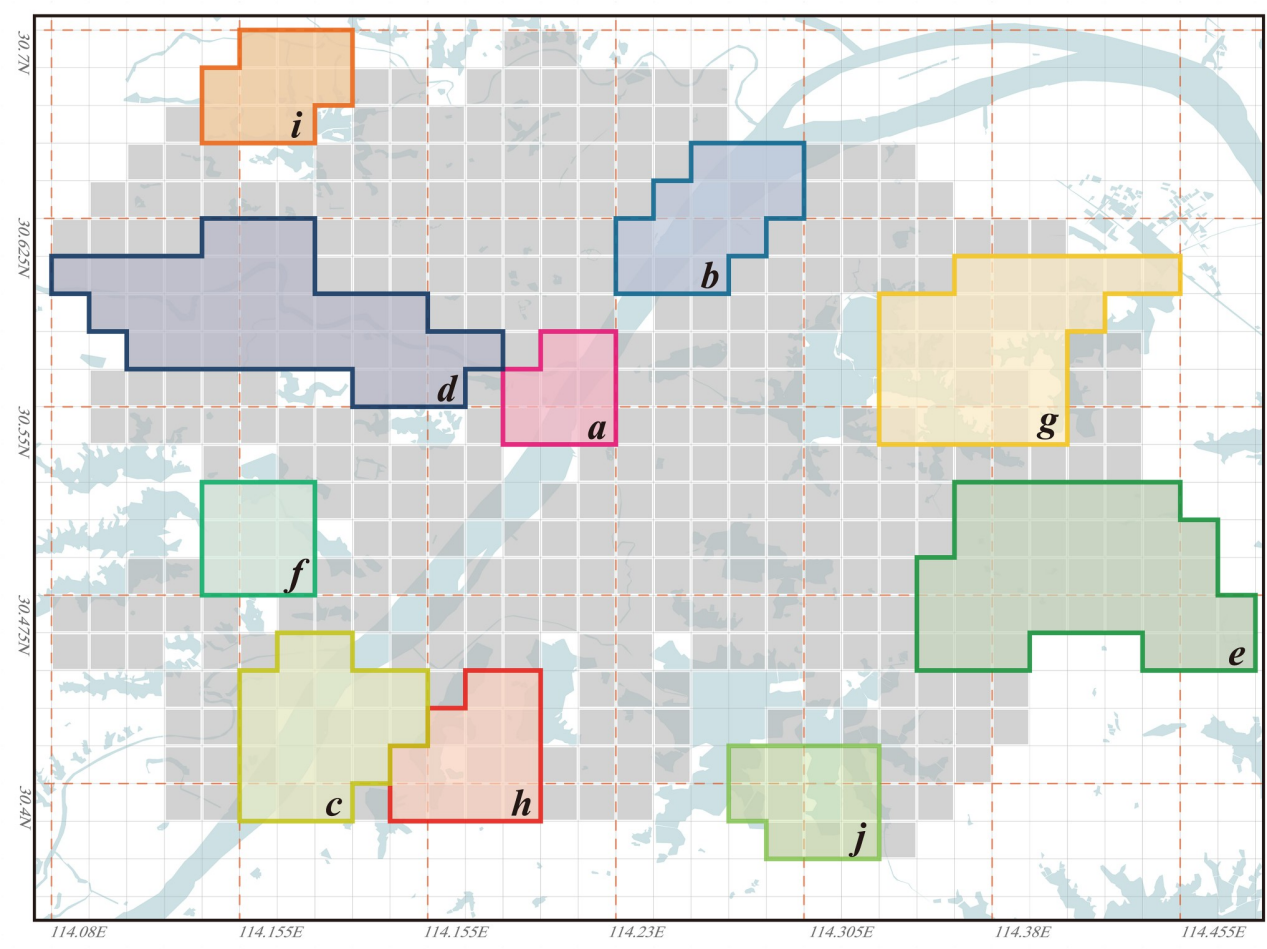
The analysis area for urban regional accessibility under the influence of water bodies in Wuhan in this study. (a) the geographic center of Wuhan urban area; (b) The northern banks of the Yangtze River within the Wuhan urban area; (c) The southern banks of the Yangtze River within the Wuhan urban area; (d) the Han River’s banks; (e) the Optics Valley area; (f) the Zhuankou area; (g) the northeastern area of East Lake; (h) the area between Huangjia Lake and Qingling Lake. Base map and data from OpenStreetMap and OpenStreetMap Foundation.

Based on the $A_{\bar{li}}$ value distribution in Wuhan for the years 1969, 1995, and 2023 as shown in Figs 9–11, the evolution characteristics of urban regional accessibility under the influence of water bodies can be summarized as follows:

1. In the years 1969, 1995, and 2023, the high values of $A_{\bar{li}}$ in Wuhan have all appeared along both sides of the Yangtze River, particularly concentrated at the junction of the three towns: Hankou, Wuchang, and Hanyang. Among them, the Nananzui of Hanyang is where the highest $A_{\bar{li}}$ value in Wuhan occurred in 1969, and this location also serves as the geographic center of Wuhan urban area (Fig 12a). Wuhan is a city that develops evenly along both sides of the Yangtze River, the Yangtze River roughly divides Wuhan into two parts. Under normal circumstances, areas in the city center tend to have the best accessibility, so the areas along both sides of the Yangtze River are commonly considered the most convenient in terms of transportation in Wuhan. However, the higher $A_{\bar{li}}$ value in this area implies that its accessibility based on its inherent environmental conditions is not ideal within the city of Wuhan, and this contradicts conventional understanding. In 1969, there was only one bridge across the Yangtze River and only one bridge across the Han River in Wuhan, so the connection between the three towns was single line. At that time, the northern areas in Hankou and the northern areas in Wuchang lacked a direct link (Fig 12b), so the $A_{\bar{li}}$ value of the two areas was obviously higher because travel between the two areas needs to detour to Hanyang. In 1969, the roads across the river in Wuhan had formed a ring, the completion of the Second Wuhan Yangtze River Bridge eased the transportation problems between the northern areas in Hankou and the northern areas in Wuchang. Therefore, the areas with the highest $A_{\bar{li}}$ value were moved to the south-central area of the city near the Yangtze River, especially the area south of Baishazhou (Fig 12c), this area is far away from the Yangtze River Bridge and the inconvenience of crossing the river is the main reason for its high $A_{\bar{li}}$ value. In 2023, the roads across the river in Wuhan had formed a network, so the distribution of $A_{\bar{li}}$ value of the nodes in the city was more even than in 1969 and 1995. However, the areas near the Yangtze River especially the intersection area of the two rivers in the center of the city were still the areas with the highest $A_{\bar{li}}$ value, and the main reason is also that the *L*_*ij*_ value for road distance/spatial distance between the nodes on different sides of the Yangtze River is too high. Differing from the years 1969 and 1995, in 2023, Wuhan’s river-crossing passages is densely distributed over the Yangtze River. However, due to the Yangtze River’s excessive width and the high bridges, the Yangtze River bridges must be equipped with long approach bridges, this significantly increases the detour distance for riverside sections during river crossing, thereby raising their $A_{\bar{li}}$ values. Among them, the vehicle access point of the Wuhan Yangtze River Bridge in Wuchang is 2.7 km away from the riverbank, this distance is the longest among all the river-crossing passages in Wuhan urban area. Consequently, in 2023, the high $A_{\bar{li}}$ value appears in the riverside section of the Yangtze River Bridge. Meanwhile, the area 1 to 2 km behind the riverside section benefits from relatively convenient river crossing due to its proximity to the entrance of the approach bridge of the Yangtze River Bridge, as a result, the $A_{\bar{li}}$ value is significantly reduced in this area. Unlike the Yangtze River’s banks, the $A_{\bar{li}}$ values along the Han River’s banks have never been excessively high (Fig 12d), indicating that their accessibility has not been significantly affected by their proximity to the river. One reason for this is that the width of the Han River is only about 1/6 of that of the Yangtze River, and the distance from the points where vehicles cross the river to the riverbank is generally only 200 to 500 meters, making the detour distance for crossing the river significantly shorter. Another reason is that the banks of the Han River are located to the west of Wuhan, away from most urban areas, so the impact of minor detours on the *L*_*ij*_ values between distant nodes is limited.
2. The Yangtze River, as the most typical “obstacle” of water body, clearly influences the road accessibility based on their inherent environmental conditions of various areas in Wuhan, and this results in consistently higher $A_{\bar{li}}$ values along both sides of the Yangtze River. This phenomenon gives rise to an interesting observation: the peripheral areas of Wuhan, far from the Yangtze River, have the lowest $A_{\bar{li}}$ values in 1969, 1995, and 2023. This suggests that although these areas are distant from the city center, their road accessibility based on their inherent environmental conditions is actually the best. Among them, in 2023, the areas with the lowest $A_{\bar{li}}$ values are mainly the Optics Valley area of Wuchang (Fig 12e) and the Zhuankou area of Hanyang (Fig 12f). The main reason for the low $A_{\bar{li}}$ value in the outer suburbs of the city is the long straight-line distance to most areas in the city, in this case, although the detour is inevitable when crossing the Yangtze River from the outer suburbs of the city, the increased detour distance is not obvious compared with the straight-line distance between the two nodes, so it is more likely to be accepted by people psychologically.
3. From the $A_{\bar{li}}$ value distribution map of 2023, it can be observed that smaller lakes in the city center have no significant impact on the accessibility of nearby nodes based on their inherent environmental conditions. Lakes with an approximate water area of 3 km², such as Sha Lake, Moshui Lake, and Longyang Lake, show no significant changes in the surrounding $A_{\bar{li}}$ values. However, larger lakes that are farther away from the city center, such as East Lake, Tangxun Lake, Huangjia Lake, Qingling Lake in Wuchang, as well as JinYin Lake in Hankou, still have an influence on the accessibility of their surrounding areas. In 2023, the $A_{\bar{li}}$ values in the northeastern area of East Lake were generally higher than 1.16 (Fig 12g), making it one of the regions with the highest $A_{\bar{li}}$ values in Wuhan, along with the area between Huangjia Lake and Qingling Lake. (Fig 12h). The reason for the extremely high $A_{\bar{li}}$ values in this area is that there is no lake-crossing passage from the northeast to the southwest leading directly to the center of Wuhan. This results in the necessity for most areas in the northeast of East Lake to take longer detours around the lake to access various areas of the city. On the other hand, the East Lake Tunnel, running from the northwest to the southeast of East Lake, was completed and opened to traffic in 2015. As a result, the $A_{\bar{li}}$ values in the tunnel entrance and exit areas have been somewhat reduced. Especially in the Optics Valley area, the East Lake Tunnel significantly shortened the transportation distance to the previously less convenient northern part of Wuchang. Consequently, the Optics Valley area has the lowest $A_{\bar{li}}$ values in Wuhan in 2023, with most nodes in this area having $A_{\bar{li}}$ values below 0.95. Furthermore, there are significant differences in the $A_{\bar{li}}$ values around large lakes such as JinYin Lake (Fig 12i) and TangXun Lake (Fig 12j). This is mainly evident in the $A_{\bar{li}}$ values on the side away from the city center being higher than those on the side closer to the city center. The primary reason for this is also the need to take longer detours around the lake to access most areas of the city on the side away from the city center. However, the $A_{\bar{li}}$ value differences around these two lakes are smaller than those around Esat Lake. The $A_{\bar{li}}$ values for nodes on the side away from the city center of these two lakes generally fall between 1.00 and 1.10.

**Fig 13:**
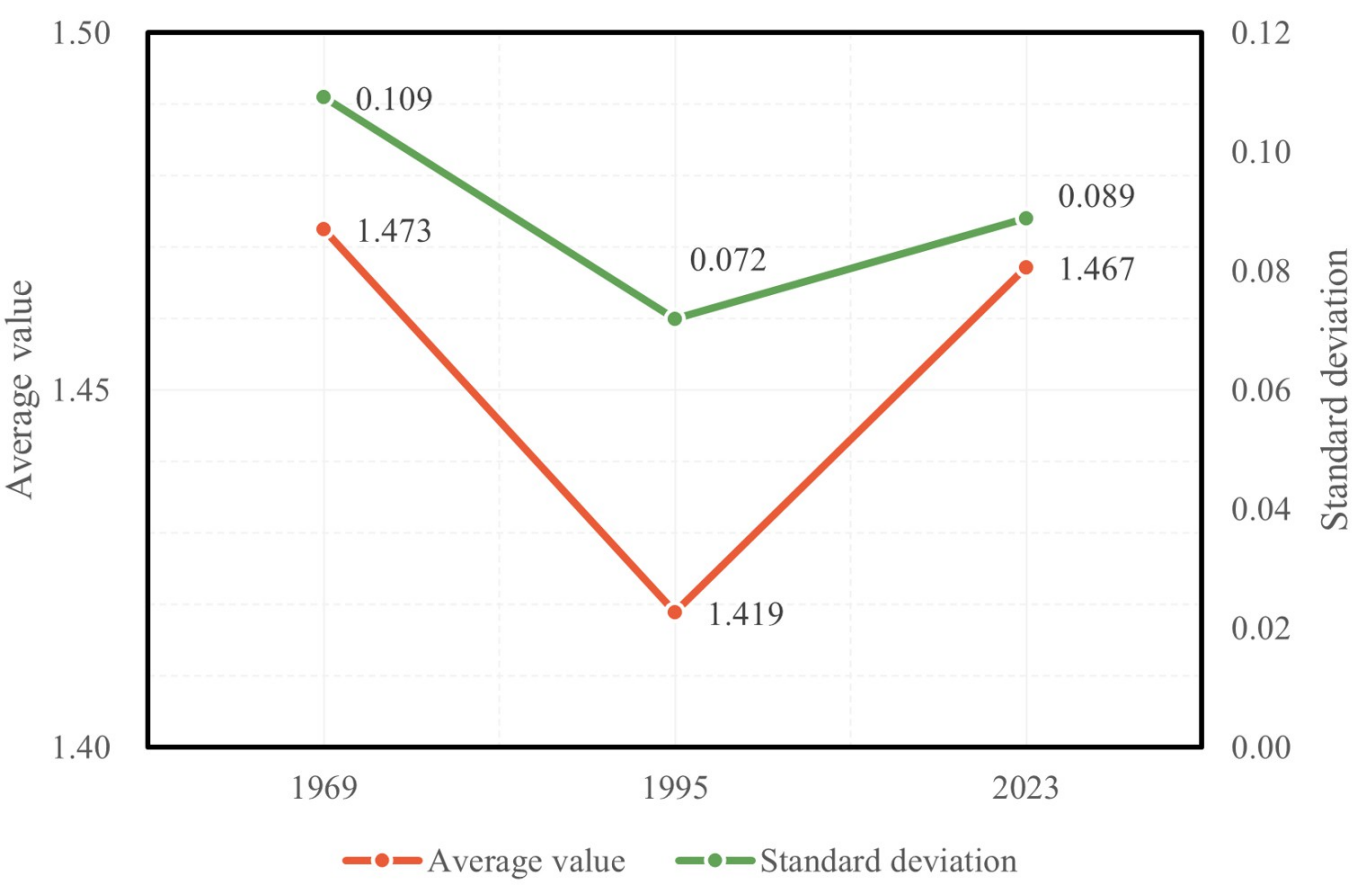
Changes in mean and standard deviation of $\bar{L_{i}}$ values for all nodes in Wuhan in 1969, 1995, and 2023.

In addition, as shown in Fig 13, the average detour index $\bar{L_{i}}$ of all nodes within Wuhan was 1.473 in 1969, decreased to 1.419 in 1995, and then increased again to 1.467 in 2023. In 1969, Wuhan had only one Yangtze River bridge and one Han River bridge, so the road across the river in Wuhan is the only. By 1995, two Yangtze River bridges and two Han River bridges had been constructed, forming a ring of the road across the river in Wuhan. During this phase, with an increase in the number of river-crossing passages and the improvement of the urban road network, the road accessibility in Wuhan had significantly improved. However, in 2023, as the city expanded further, even though the road network in Wuhan was already well-developed and the city had multiple river-crossing and lake-crossing passages, some large lakes that were originally located outside the city had become part of the urban area. The inadequate road accessibility around these lakes due to the influence of water bodies resulted in a decrease in the overall road accessibility of the city. On the other hand, the standard deviation of the detour index $\bar{L_{i}}$ for all nodes within Wuhan was 0.109 in 1969, decreased substantially to 0.072 in 1995, and then increased to 0.089 in 2023. It can be seen that in the early stages when there were insufficient river-crossing passages, there were significant differences in $\bar{L_{i}}$ values among different areas of the city. With an increase in the number of river-crossing passages and the improvement of the urban road network, the distribution of $\bar{L_{i}}$ values in various areas of Wuhan became more balanced. However, as mentioned earlier, due to the poor road accessibility based on its inherent environmental conditions around large lakes on the periphery of Wuhan after the city expanded, and with $\bar{L_{i}}$ values significantly different from the majority of areas with good road networks within the city, this resulted in an increased disparity in road accessibility based on its inherent environmental conditions among different areas of the city.

## Conclusions, deficiencies, and prospects

In the past century, rivers and lakes have had a profound impact on the urban expansion of Wuhan. They have gone through different stages, such as “strong connection”, “weakened connection” and “mutual restriction”. What is currently needed is the mutual development of both. In the process of urban expansion in Wuhan over the past century, the connection between rivers and transportation has weakened, while the connection between lakes and transportation has continued to strengthen. Lakes that were once located outside the city have gradually become located within the urban area. This phenomenon is also common in the expansion of many water-rich cities in China, such as Jinji Lake, Dushu Lake, and Yangcheng Lake in Suzhou, Taihu Lake in Wuxi, Ge Lake in Changzhou, and Chaohu Lake in Hefei, among others. Therefore, how to better consider lakeside and lake-crossing transportation in urban road network planning, and how to protect the relatively fragile ecological environment of urban lakes more reasonably on this basis, will be an important research direction for the relationship between water bodies and urban transportation in the future.

In addition, based on the research in this paper, the following conclusions can be drawn:

1. Rivers running through the city center act as “obstacles” in the urban road network, leading to increased overall detour degree in the city and affecting urban expansion. This is particularly evident when the rivers are wide, and the number of river-crossing passages is limited. In the river-span cities, even if the riverbanks are located in the city center, their detour index remains much higher than other areas of the city. This indicates that the road accessibility based on their inherent environmental conditions of the riverbanks may be poor, potentially making the riverbanks, despite being the geographical center of the city, a terminal in urban transportation. In such a scenario, if the city’s major industries are concentrated along the riverbanks and seek coordination and integration to create a synergy, the convenience of transportation between the two riverbanks may fall below the expectations of the population. Therefore, urban transportation development and industrial layout should fully consider the physical characteristics of rivers as typical “obstacles” in the urban road network and incorporate mitigation strategies into the city’s planning.
2. Large lakes are also significant “obstacles” within urban road networks. This is manifested in areas near these lakes, particularly those located away from the city center, where road accessibility based on their inherent environmental conditions is poor. Moreover, the larger the lake, the more pronounced this characteristic becomes. When bridges or tunnels are constructed across lakes, the detour indices at their entrances and exits will significantly decrease. However, for larger lakes, a single bridge or tunnel has limited effectiveness in improving road accessibility based on their inherent environmental conditions of the lakefront areas. Instead, it may potentially harm the ecological environment of the lake. Areas far from the entrances and exits of the bridge or tunnel cannot achieve a significant improvement in road accessibility based on their inherent environmental conditions as a result. The aforementioned road accessibility issues also present challenges for urban development along lakeshores.
3. For cities with lots of water, the improvement of the urban road network and the increased number of river-crossing and lake-crossing passages can effectively enhance the overall road accessibility of the city. Additionally, it can promote a more balanced road accessibility based on their inherent environmental conditions in different areas of the city. However, as the city continues to expand, large water bodies that were originally located outside the city may become part of the urban area. The road accessibility based on their inherent environmental conditions in the surrounding areas of these water bodies tends to be poor, leading to a reduction in the overall road accessibility of the urban road network and an increase in the disparities in road accessibility based on their inherent environmental conditions among different areas of the city. This is a point that urban planners and administrators often overlook. Therefore, when planning urban road networks, special attention should be given to the development of transportation infrastructure around the water bodies at the periphery of the city, ensuring that it keeps pace with the urban development and industrial layout in that region as much as possible.

Based on the above conclusions, in the pursuit of mutual benefits between urban water bodies and urban transportation, attention should be given to the inherent deficiencies in the accessibility of roads along rivers and lakes. Emphasis should be placed on the construction of slow traffic systems, such as urban greenways, based on reasonable land use planning. This approach fully leverages the ecological advantages of urban areas along rivers and lakes, aiming to avoid further disruption to the ecological balance caused by the additional strengthening of transportation infrastructure construction in these areas due to inadequate accessibility.

The main contribution of this paper lies in proposing, for the first time based on buffer analysis, the developmental relationship between water bodies and urban transportation, characterized by “strong connection—weakened connection—mutual restriction—mutual benefit.” Building upon this, the paper introduces the concepts of “detour index” and “weighted detour index” by modifying the traditional shortest path model. These concepts are used to assess the road accessibility of urban nodes based on their inherent environmental conditions. The method eliminates the consideration of location advantages in assessing urban road accessibility, focusing on the real impact of external environmental characteristics on accessibility. It aligns with the analysis of the impact of water bodies on the evolution of urban road networks. Furthermore, it can be applied in future research on the impact of factors such as topography, built environment, and other elements on urban road network accessibility. This research proposes the concepts of “Detour Index” and “Weighted Detour Index” to assess the impact of water bodies as “obstacles” on urban transportation accessibility, but these concepts consider only completely objective distance factors. However, urban residents have diverse modes of transportation, including walking, cycling, public transit, and driving, each of which may exhibit different detour characteristics. The subjective impact of water bodies as “obstacles” on people’s travel behavior is closely related to urban transportation accessibility. Therefore, it should also be included in the research scope of the relationship between urban water bodies and urban transportation. The shortcomings mentioned above will be the focus of our next steps in research.

## Supporting information

S1 File Containing S1-S3 Tables. The Impact of Rivers and Lakes on Urban Transportation Expansion: A Case Study of the Century-Long Evolution of the Road Network in Wuhan, China.
(DOCX)
