# Supplementary material for: The impact of rivers and lakes on urban transportation expansion: A case study of the century-long evolution of the road network in Wuhan, China
Source: PLoS One. 2024 Mar 18;19(3):e0298678. doi: 10.1371/journal.pone.0298678 (PMC10947703; doi:10.1371/journal.pone.0298678)
Supplement: S1 File — The Impact of Rivers and Lakes on Urban Transportation Expansion: A Case Study of the Century-Long Evolution of the Road Network in Wuhan, China. (DOCX) [file pone.0298678.s001.docx]

The Impact of Rivers and Lakes on Urban Transportation Expansion: A Case Study of the Century-Long Evolution of the Road Network in Wuhan, China

**Table S1.** $\bar{\boldsymbol{L}_{\boldsymbol{l}}}$ **and** $\boldsymbol{A}_{\bar{\boldsymbol{li}}}$ **values for all nodes in Wuhan in 1969，presented in Fig 6，9.**

| **Coordinates of the Central**  **Point of nodes** | $\bar{\boldsymbol{L}_{\boldsymbol{l}}}$ **Values** | $\boldsymbol{A}_{\bar{\boldsymbol{li}}}$ **Values** |
| --- | --- | --- |
| (114.1925E,30.5875N) | 1.354718 | 0.919973 |
| (114.2075E,30.5875N) | 1.353784 | 0.919339 |
| (114.2075E,30.6025N) | 1.301998 | 0.884172 |
| (114.2225E,30.5575N) | 1.417337 | 0.962497 |
| (114.2225E,30.5725N) | 1.449329 | 0.984222 |
| (114.2225E,30.5875N) | 1.425902 | 0.968313 |
| (114.2225E,30.6025N) | 1.53497 | 1.04238 |
| (114.2375E,30.5575N) | 1.433037 | 0.973158 |
| (114.2375E,30.5725N) | 1.485517 | 1.008797 |
| (114.2375E,30.5875N) | 1.529412 | 1.038606 |
| (114.2375E,30.6025N) | 1.463285 | 0.9937 |
| (114.2525E,30.5275N) | 1.47766 | 1.003461 |
| (114.2525E,30.5425N) | 1.485605 | 1.008857 |
| (114.2525E,30.5575N) | 1.35282 | 0.918684 |
| (114.2525E,30.5725N) | 1.445193 | 0.981414 |
| (114.2525E,30.5875N) | 1.521893 | 1.0335 |
| (114.2525E,30.6025N) | 1.506887 | 1.023309 |
| (114.2525E,30.6175N) | 1.307238 | 0.88773 |
| (114.2675E,30.5425N) | 1.533236 | 1.041203 |
| (114.2675E,30.5575N) | 1.512229 | 1.026937 |
| (114.2675E,30.5725N) | 1.422396 | 0.965933 |
| (114.2675E,30.5875N) | 1.469786 | 0.998115 |
| (114.2675E,30.6025N) | 1.431743 | 0.97228 |
| (114.2675E,30.6175N) | 1.395284 | 0.947521 |
| (114.2825E,30.5125N) | 1.557803 | 1.057885 |
| (114.2825E,30.5575N) | 1.764003 | 1.197914 |
| (114.2825E,30.5725N) | 1.525692 | 1.036079 |
| (114.2825E,30.5875N) | 1.472529 | 0.999977 |
| (114.2825E,30.6025N) | 1.419587 | 0.964025 |
| (114.2825E,30.6175N) | 1.35697 | 0.921503 |
| (114.2975E,30.5125N) | 1.449683 | 0.984463 |
| (114.2975E,30.5275N) | 1.457919 | 0.990056 |
| (114.2975E,30.5425N) | 1.400176 | 0.950843 |
| (114.2975E,30.5575N) | 1.568093 | 1.064873 |
| (114.2975E,30.5875N) | 1.507028 | 1.023405 |
| (114.2975E,30.6025N) | 1.440291 | 0.978085 |
| (114.2975E,30.6175N) | 1.443939 | 0.980562 |
| (114.2975E,30.6325N) | 1.534798 | 1.042263 |
| (114.3125E,30.5125N) | 1.454251 | 0.987565 |
| (114.3125E,30.5275N) | 1.488532 | 1.010845 |
| (114.3125E,30.5425N) | 1.435446 | 0.974795 |
| (114.3125E,30.5575N) | 1.456738 | 0.989254 |
| (114.3125E,30.5725N) | 1.463542 | 0.993874 |
| (114.3125E,30.6025N) | 1.59918 | 1.085985 |
| (114.3125E,30.6175N) | 1.458617 | 0.99053 |
| (114.3125E,30.6325N) | 1.409091 | 0.956897 |
| (114.3125E,30.6475N) | 1.44595 | 0.981927 |
| (114.3275E,30.5275N) | 1.392005 | 0.945294 |
| (114.3275E,30.5425N) | 1.354002 | 0.919487 |
| (114.3275E,30.5875N) | 1.492229 | 1.013355 |
| (114.3275E,30.6025N) | 1.529183 | 1.03845 |
| (114.3275E,30.6325N) | 1.528969 | 1.038305 |
| (114.3275E,30.6475N) | 1.555165 | 1.056095 |
| (114.3275E,30.6625N) | 1.449294 | 0.984199 |
| (114.3425E,30.5275N) | 1.411633 | 0.958623 |
| (114.3425E,30.5425N) | 1.440484 | 0.978216 |
| (114.3425E,30.5575N) | 1.430701 | 0.971573 |
| (114.3425E,30.6025N) | 1.478517 | 1.004044 |
| (114.3425E,30.6175N) | 1.513399 | 1.027732 |
| (114.3425E,30.6475N) | 2.042307 | 1.386907 |
| (114.3425E,30.6625N) | 1.469473 | 0.997902 |
| (114.3575E,30.5275N) | 1.372179 | 0.931831 |
| (114.3575E,30.5425N) | 1.511779 | 1.026631 |
| (114.3575E,30.5575N) | 1.665986 | 1.131352 |
| (114.3575E,30.6025N) | 1.40311 | 0.952836 |
| (114.3575E,30.6175N) | 1.976169 | 1.341993 |
| (114.3575E,30.6325N) | 1.564398 | 1.062364 |
| (114.3725E,30.5125N) | 1.404297 | 0.953642 |
| (114.3725E,30.5275N) | 1.519614 | 1.031952 |
| (114.3725E,30.5725N) | 1.451514 | 0.985706 |
| (114.3725E,30.5875N) | 1.510822 | 1.025982 |
| (114.3725E,30.6025N) | 1.424435 | 0.967317 |
| (114.3725E,30.6175N) | 1.514304 | 1.028346 |
| (114.3725E,30.6325N) | 1.471613 | 0.999355 |
| (114.3875E,30.4975N) | 1.444951 | 0.981249 |
| (114.3875E,30.5125N) | 1.511917 | 1.026725 |
| (114.3875E,30.5875N) | 1.514104 | 1.028211 |
| (114.3875E,30.6025N) | 1.527862 | 1.037553 |
| (114.3875E,30.6175N) | 1.50306 | 1.020711 |
| (114.3875E,30.6325N) | 1.386106 | 0.941288 |
| (114.3875E,30.6475N) | 1.358973 | 0.922863 |
| (114.4025E,30.4975N) | 1.354613 | 0.919902 |
| (114.4025E,30.5125N) | 1.464789 | 0.994721 |
| (114.4025E,30.6025N) | 1.458588 | 0.99051 |
| (114.4025E,30.6175N) | 1.41966 | 0.964075 |
| (114.4025E,30.6325N) | 1.353225 | 0.918959 |
| (114.4025E,30.6475N) | 1.354183 | 0.91961 |
| (114.4175E,30.5125N) | 1.405319 | 0.954336 |
| (114.4175E,30.6025N) | 1.409313 | 0.957048 |
| (114.4175E,30.6175N) | 1.648884 | 1.119738 |
| (114.4175E,30.6325N) | 1.350201 | 0.916906 |
| (114.4175E,30.6475N) | 1.384915 | 0.940479 |
| (114.4325E,30.6025N) | 1.58779 | 1.07825 |
| (114.4325E,30.6175N) | 1.456033 | 0.988775 |
| (114.4325E,30.6325N) | 1.508925 | 1.024693 |
| (114.4475E,30.6025N) | 1.451527 | 0.985715 |
| (114.4475E,30.6175N) | 1.384931 | 0.94049 |

**Table S2.** $\bar{\boldsymbol{L}_{\boldsymbol{l}}}$ **and** $\boldsymbol{A}_{\bar{\boldsymbol{li}}}$ **values for all nodes in Wuhan in 1995，presented in Fig 7，10.**

| **Coordinates of the Central**  **Point of nodes** | $\bar{\boldsymbol{L}_{\boldsymbol{l}}}$ **Values** | $\boldsymbol{A}_{\bar{\boldsymbol{li}}}$ **Values** |
| --- | --- | --- |
| (114.1175E,30.4675N) | 1.280512 | 0.90246 |
| (114.1175E,30.4825N) | 1.337503 | 0.942626 |
| (114.1175E,30.6175N) | 1.387903 | 0.978146 |
| (114.1175E,30.6325N) | 1.38922 | 0.979074 |
| (114.1325E,30.4675N) | 1.337553 | 0.942661 |
| (114.1325E,30.4825N) | 1.334091 | 0.940221 |
| (114.1325E,30.6025N) | 1.383859 | 0.975296 |
| (114.1325E,30.6175N) | 1.364749 | 0.961828 |
| (114.1325E,30.6325N) | 1.408967 | 0.992991 |
| (114.1475E,30.4675N) | 1.393831 | 0.982324 |
| (114.1475E,30.4825N) | 1.310541 | 0.923624 |
| (114.1475E,30.4975N) | 1.365702 | 0.9625 |
| (114.1475E,30.6025N) | 1.393284 | 0.981938 |
| (114.1475E,30.6175N) | 1.360096 | 0.958548 |
| (114.1475E,30.6325N) | 1.449746 | 1.02173 |
| (114.1625E,30.4825N) | 1.393611 | 0.982168 |
| (114.1625E,30.4975N) | 1.371286 | 0.966435 |
| (114.1625E,30.5125N) | 1.411705 | 0.994921 |
| (114.1625E,30.6175N) | 1.355988 | 0.955653 |
| (114.1625E,30.6325N) | 1.46763 | 1.034335 |
| (114.1775E,30.4675N) | 1.372011 | 0.966945 |
| (114.1775E,30.4825N) | 1.434313 | 1.010854 |
| (114.1775E,30.4975N) | 1.438899 | 1.014086 |
| (114.1775E,30.5125N) | 1.37053 | 0.965902 |
| (114.1775E,30.5275N) | 1.404864 | 0.990099 |
| (114.1775E,30.5425N) | 1.341649 | 0.945547 |
| (114.1775E,30.5875N) | 1.396012 | 0.983861 |
| (114.1775E,30.6025N) | 1.391543 | 0.980711 |
| (114.1775E,30.6175N) | 1.40516 | 0.990308 |
| (114.1775E,30.6325N) | 1.425459 | 1.004614 |
| (114.1925E,30.4675N) | 1.396561 | 0.984248 |
| (114.1925E,30.4825N) | 1.368459 | 0.964443 |
| (114.1925E,30.5125N) | 1.405802 | 0.99076 |
| (114.1925E,30.5275N) | 1.408907 | 0.992949 |
| (114.1925E,30.5425N) | 1.333662 | 0.939918 |
| (114.1925E,30.5725N) | 1.334438 | 0.940466 |
| (114.1925E,30.5875N) | 1.448666 | 1.02097 |
| (114.1925E,30.6025N) | 1.36909 | 0.964887 |
| (114.1925E,30.6175N) | 1.394446 | 0.982757 |
| (114.2075E,30.4675N) | 1.375889 | 0.969679 |
| (114.2075E,30.4825N) | 1.489417 | 1.04969 |
| (114.2075E,30.4975N) | 1.414804 | 0.997105 |
| (114.2075E,30.5125N) | 1.393347 | 0.981983 |
| (114.2075E,30.5275N) | 1.41185 | 0.995023 |
| (114.2075E,30.5425N) | 1.408402 | 0.992593 |
| (114.2075E,30.5575N) | 1.34963 | 0.951172 |
| (114.2075E,30.5725N) | 1.366756 | 0.963242 |
| (114.2075E,30.5875N) | 1.386329 | 0.977036 |
| (114.2075E,30.6025N) | 1.357728 | 0.95688 |
| (114.2225E,30.4825N) | 1.39861 | 0.985692 |
| (114.2225E,30.4975N) | 1.459048 | 1.028287 |
| (114.2225E,30.5125N) | 1.422777 | 1.002724 |
| (114.2225E,30.5275N) | 1.481445 | 1.044071 |
| (114.2225E,30.5575N) | 1.394803 | 0.983009 |
| (114.2225E,30.5725N) | 1.416016 | 0.997959 |
| (114.2225E,30.5875N) | 1.403395 | 0.989064 |
| (114.2225E,30.6025N) | 1.482865 | 1.045072 |
| (114.2225E,30.6175N) | 1.547601 | 1.090696 |
| (114.2225E,30.6325N) | 1.400333 | 0.986906 |
| (114.2375E,30.4975N) | 1.529783 | 1.078138 |
| (114.2375E,30.5125N) | 1.485956 | 1.047251 |
| (114.2375E,30.5275N) | 1.446133 | 1.019185 |
| (114.2375E,30.5575N) | 1.411194 | 0.994561 |
| (114.2375E,30.5725N) | 1.434429 | 1.010936 |
| (114.2375E,30.5875N) | 1.495098 | 1.053693 |
| (114.2375E,30.6025N) | 1.4477 | 1.020289 |
| (114.2375E,30.6175N) | 1.429804 | 1.007676 |
| (114.2375E,30.6325N) | 1.353487 | 0.953891 |
| (114.2375E,30.6475N) | 1.385714 | 0.976603 |
| (114.2525E,30.4825N) | 1.679141 | 1.1834 |
| (114.2525E,30.5125N) | 1.565469 | 1.103288 |
| (114.2525E,30.5275N) | 1.476415 | 1.040526 |
| (114.2525E,30.5425N) | 1.447942 | 1.02046 |
| (114.2525E,30.5575N) | 1.380668 | 0.973047 |
| (114.2525E,30.5725N) | 1.391381 | 0.980597 |
| (114.2525E,30.5875N) | 1.427057 | 1.00574 |
| (114.2525E,30.6025N) | 1.423765 | 1.00342 |
| (114.2525E,30.6175N) | 1.314279 | 0.926258 |
| (114.2525E,30.6325N) | 1.336971 | 0.942251 |
| (114.2675E,30.4825N) | 1.506111 | 1.061455 |
| (114.2675E,30.4975N) | 1.524961 | 1.07474 |
| (114.2675E,30.5425N) | 1.470392 | 1.036281 |
| (114.2675E,30.5575N) | 1.403815 | 0.98936 |
| (114.2675E,30.5725N) | 1.3631 | 0.960666 |
| (114.2675E,30.5875N) | 1.402877 | 0.988699 |
| (114.2675E,30.6025N) | 1.378156 | 0.971277 |
| (114.2675E,30.6175N) | 1.378231 | 0.971329 |
| (114.2675E,30.6325N) | 1.373828 | 0.968226 |
| (114.2675E,30.6475N) | 1.332366 | 0.939005 |
| (114.2825E,30.4975N) | 1.434098 | 1.010703 |
| (114.2825E,30.5125N) | 1.755952 | 1.237534 |
| (114.2825E,30.5575N) | 1.592212 | 1.122136 |
| (114.2825E,30.5725N) | 1.458689 | 1.028033 |
| (114.2825E,30.5875N) | 1.399653 | 0.986427 |
| (114.2825E,30.6025N) | 1.361618 | 0.959621 |
| (114.2825E,30.6175N) | 1.326225 | 0.934677 |
| (114.2825E,30.6325N) | 1.317284 | 0.928376 |
| (114.2975E,30.4975N) | 1.492816 | 1.052085 |
| (114.2975E,30.5125N) | 1.475771 | 1.040073 |
| (114.2975E,30.5275N) | 1.46458 | 1.032185 |
| (114.2975E,30.5425N) | 1.466918 | 1.033833 |
| (114.2975E,30.5575N) | 1.615371 | 1.138457 |
| (114.2975E,30.5875N) | 1.447911 | 1.020438 |
| (114.2975E,30.6025N) | 1.346706 | 0.949112 |
| (114.2975E,30.6175N) | 1.370002 | 0.96553 |
| (114.2975E,30.6325N) | 1.456284 | 1.026338 |
| (114.2975E,30.6475N) | 1.416015 | 0.997958 |
| (114.3125E,30.4975N) | 1.457722 | 1.027352 |
| (114.3125E,30.5125N) | 1.463277 | 1.031267 |
| (114.3125E,30.5275N) | 1.464561 | 1.032172 |
| (114.3125E,30.5425N) | 1.44008 | 1.014918 |
| (114.3125E,30.5575N) | 1.468645 | 1.03505 |
| (114.3125E,30.5725N) | 1.48989 | 1.050023 |
| (114.3125E,30.6025N) | 1.475549 | 1.039916 |
| (114.3125E,30.6175N) | 1.381481 | 0.97362 |
| (114.3125E,30.6325N) | 1.3508 | 0.951997 |
| (114.3125E,30.6475N) | 1.358815 | 0.957646 |
| (114.3275E,30.5125N) | 1.475472 | 1.039861 |
| (114.3275E,30.5275N) | 1.415794 | 0.997802 |
| (114.3275E,30.5425N) | 1.395756 | 0.98368 |
| (114.3275E,30.5725N) | 1.442794 | 1.016832 |
| (114.3275E,30.5875N) | 1.455545 | 1.025818 |
| (114.3275E,30.6025N) | 1.494198 | 1.053059 |
| (114.3275E,30.6325N) | 1.391467 | 0.980658 |
| (114.3275E,30.6475N) | 1.379717 | 0.972377 |
| (114.3275E,30.6625N) | 1.343067 | 0.946547 |
| (114.3275E,30.6775N) | 1.313862 | 0.925965 |
| (114.3425E,30.5125N) | 1.402765 | 0.98862 |
| (114.3425E,30.5275N) | 1.41778 | 0.999202 |
| (114.3425E,30.5425N) | 1.408268 | 0.992499 |
| (114.3425E,30.5575N) | 1.440809 | 1.015432 |
| (114.3425E,30.5875N) | 1.444147 | 1.017785 |
| (114.3425E,30.6025N) | 1.418705 | 0.999854 |
| (114.3425E,30.6175N) | 1.452914 | 1.023963 |
| (114.3425E,30.6475N) | 1.542425 | 1.087048 |
| (114.3425E,30.6625N) | 1.327659 | 0.935688 |
| (114.3425E,30.6775N) | 1.318383 | 0.929151 |
| (114.3575E,30.5275N) | 1.434839 | 1.011225 |
| (114.3575E,30.5425N) | 1.448558 | 1.020894 |
| (114.3575E,30.5575N) | 1.764263 | 1.243392 |
| (114.3575E,30.5725N) | 1.42651 | 1.005355 |
| (114.3575E,30.6025N) | 1.376593 | 0.970175 |
| (114.3575E,30.6175N) | 1.54069 | 1.085825 |
| (114.3575E,30.6325N) | 1.362576 | 0.960296 |
| (114.3725E,30.4825N) | 1.477957 | 1.041613 |
| (114.3725E,30.5125N) | 1.392282 | 0.981232 |
| (114.3725E,30.5275N) | 1.449151 | 1.021311 |
| (114.3725E,30.5425N) | 1.425879 | 1.00491 |
| (114.3725E,30.5575N) | 1.48767 | 1.048458 |
| (114.3725E,30.5725N) | 1.413915 | 0.996478 |
| (114.3725E,30.5875N) | 1.458282 | 1.027746 |
| (114.3725E,30.6025N) | 1.425294 | 1.004498 |
| (114.3725E,30.6175N) | 1.361044 | 0.959216 |
| (114.3725E,30.6325N) | 1.389309 | 0.979137 |
| (114.3875E,30.4675N) | 1.445644 | 1.018839 |
| (114.3875E,30.4825N) | 1.429203 | 1.007253 |
| (114.3875E,30.4975N) | 1.39799 | 0.985255 |
| (114.3875E,30.5125N) | 1.476666 | 1.040703 |
| (114.3875E,30.5275N) | 1.52513 | 1.074859 |
| (114.3875E,30.5875N) | 1.4469 | 1.019725 |
| (114.3875E,30.6025N) | 1.472401 | 1.037697 |
| (114.3875E,30.6175N) | 1.423426 | 1.003181 |
| (114.3875E,30.6325N) | 1.335365 | 0.941119 |
| (114.3875E,30.6475N) | 1.334402 | 0.94044 |
| (114.4025E,30.4675N) | 1.420327 | 1.000997 |
| (114.4025E,30.4825N) | 1.372383 | 0.967208 |
| (114.4025E,30.4975N) | 1.349865 | 0.951338 |
| (114.4025E,30.5125N) | 1.44324 | 1.017146 |
| (114.4025E,30.5275N) | 1.52534 | 1.075007 |
| (114.4025E,30.6025N) | 1.464113 | 1.031856 |
| (114.4025E,30.6175N) | 1.388474 | 0.978548 |
| (114.4025E,30.6325N) | 1.338586 | 0.943389 |
| (114.4025E,30.6475N) | 1.317148 | 0.92828 |
| (114.4175E,30.4675N) | 1.512537 | 1.065983 |
| (114.4175E,30.4825N) | 1.334842 | 0.940751 |
| (114.4175E,30.4975N) | 1.516084 | 1.068483 |
| (114.4175E,30.5125N) | 1.39527 | 0.983338 |
| (114.4175E,30.5275N) | 1.491769 | 1.051347 |
| (114.4175E,30.6025N) | 1.378696 | 0.971657 |
| (114.4175E,30.6175N) | 1.533602 | 1.08083 |
| (114.4175E,30.6325N) | 1.330303 | 0.937551 |
| (114.4175E,30.6475N) | 1.342967 | 0.946477 |
| (114.4325E,30.4825N) | 1.318593 | 0.929298 |
| (114.4325E,30.4975N) | 1.360781 | 0.959032 |
| (114.4325E,30.5125N) | 1.390522 | 0.979992 |
| (114.4325E,30.6025N) | 1.535403 | 1.082099 |
| (114.4325E,30.6175N) | 1.399825 | 0.986548 |
| (114.4325E,30.6325N) | 1.441352 | 1.015815 |
| (114.4475E,30.4825N) | 1.330545 | 0.937722 |
| (114.4475E,30.4975N) | 1.307095 | 0.921195 |
| (114.4475E,30.6025N) | 1.496932 | 1.054986 |
| (114.4475E,30.6175N) | 1.378422 | 0.971464 |
| (114.4625E,30.6175N) | 1.387095 | 0.977577 |
| (114.1925E,30.5875N) | 1.354718 | 0.919973 |
| (114.2075E,30.5875N) | 1.353784 | 0.919339 |
| (114.2075E,30.6025N) | 1.301998 | 0.884172 |
| (114.2225E,30.5575N) | 1.417337 | 0.962497 |
| (114.2225E,30.5725N) | 1.449329 | 0.984222 |
| (114.2225E,30.5875N) | 1.425902 | 0.968313 |
| (114.2225E,30.6025N) | 1.53497 | 1.04238 |
| (114.2375E,30.5575N) | 1.433037 | 0.973158 |
| (114.2375E,30.5725N) | 1.485517 | 1.008797 |
| (114.2375E,30.5875N) | 1.529412 | 1.038606 |
| (114.2375E,30.6025N) | 1.463285 | 0.9937 |
| (114.2525E,30.5275N) | 1.47766 | 1.003461 |
| (114.2525E,30.5425N) | 1.485605 | 1.008857 |
| (114.2525E,30.5575N) | 1.35282 | 0.918684 |
| (114.2525E,30.5725N) | 1.445193 | 0.981414 |
| (114.2525E,30.5875N) | 1.521893 | 1.0335 |
| (114.2525E,30.6025N) | 1.506887 | 1.023309 |
| (114.2525E,30.6175N) | 1.307238 | 0.88773 |
| (114.2675E,30.5425N) | 1.533236 | 1.041203 |
| (114.2675E,30.5575N) | 1.512229 | 1.026937 |
| (114.2675E,30.5725N) | 1.422396 | 0.965933 |
| (114.2675E,30.5875N) | 1.469786 | 0.998115 |
| (114.2675E,30.6025N) | 1.431743 | 0.97228 |
| (114.2675E,30.6175N) | 1.395284 | 0.947521 |
| (114.2825E,30.5125N) | 1.557803 | 1.057885 |
| (114.2825E,30.5575N) | 1.764003 | 1.197914 |
| (114.2825E,30.5725N) | 1.525692 | 1.036079 |
| (114.2825E,30.5875N) | 1.472529 | 0.999977 |
| (114.2825E,30.6025N) | 1.419587 | 0.964025 |
| (114.2825E,30.6175N) | 1.35697 | 0.921503 |
| (114.2975E,30.5125N) | 1.449683 | 0.984463 |
| (114.2975E,30.5275N) | 1.457919 | 0.990056 |
| (114.2975E,30.5425N) | 1.400176 | 0.950843 |
| (114.2975E,30.5575N) | 1.568093 | 1.064873 |
| (114.2975E,30.5875N) | 1.507028 | 1.023405 |
| (114.2975E,30.6025N) | 1.440291 | 0.978085 |
| (114.2975E,30.6175N) | 1.443939 | 0.980562 |
| (114.2975E,30.6325N) | 1.534798 | 1.042263 |
| (114.3125E,30.5125N) | 1.454251 | 0.987565 |
| (114.3125E,30.5275N) | 1.488532 | 1.010845 |
| (114.3125E,30.5425N) | 1.435446 | 0.974795 |
| (114.3125E,30.5575N) | 1.456738 | 0.989254 |
| (114.3125E,30.5725N) | 1.463542 | 0.993874 |
| (114.3125E,30.6025N) | 1.59918 | 1.085985 |
| (114.3125E,30.6175N) | 1.458617 | 0.99053 |
| (114.3125E,30.6325N) | 1.409091 | 0.956897 |
| (114.3125E,30.6475N) | 1.44595 | 0.981927 |
| (114.3275E,30.5275N) | 1.392005 | 0.945294 |
| (114.3275E,30.5425N) | 1.354002 | 0.919487 |
| (114.3275E,30.5875N) | 1.492229 | 1.013355 |
| (114.3275E,30.6025N) | 1.529183 | 1.03845 |
| (114.3275E,30.6325N) | 1.528969 | 1.038305 |
| (114.3275E,30.6475N) | 1.555165 | 1.056095 |
| (114.3275E,30.6625N) | 1.449294 | 0.984199 |
| (114.3425E,30.5275N) | 1.411633 | 0.958623 |
| (114.3425E,30.5425N) | 1.440484 | 0.978216 |
| (114.3425E,30.5575N) | 1.430701 | 0.971573 |
| (114.3425E,30.6025N) | 1.478517 | 1.004044 |
| (114.3425E,30.6175N) | 1.513399 | 1.027732 |
| (114.3425E,30.6475N) | 2.042307 | 1.386907 |
| (114.3425E,30.6625N) | 1.469473 | 0.997902 |
| (114.3575E,30.5275N) | 1.372179 | 0.931831 |
| (114.3575E,30.5425N) | 1.511779 | 1.026631 |
| (114.3575E,30.5575N) | 1.665986 | 1.131352 |
| (114.3575E,30.6025N) | 1.40311 | 0.952836 |
| (114.3575E,30.6175N) | 1.976169 | 1.341993 |
| (114.3575E,30.6325N) | 1.564398 | 1.062364 |
| (114.3725E,30.5125N) | 1.404297 | 0.953642 |
| (114.3725E,30.5275N) | 1.519614 | 1.031952 |
| (114.3725E,30.5725N) | 1.451514 | 0.985706 |
| (114.3725E,30.5875N) | 1.510822 | 1.025982 |
| (114.3725E,30.6025N) | 1.424435 | 0.967317 |
| (114.3725E,30.6175N) | 1.514304 | 1.028346 |
| (114.3725E,30.6325N) | 1.471613 | 0.999355 |
| (114.3875E,30.4975N) | 1.444951 | 0.981249 |
| (114.3875E,30.5125N) | 1.511917 | 1.026725 |
| (114.3875E,30.5875N) | 1.514104 | 1.028211 |
| (114.3875E,30.6025N) | 1.527862 | 1.037553 |
| (114.3875E,30.6175N) | 1.50306 | 1.020711 |
| (114.3875E,30.6325N) | 1.386106 | 0.941288 |
| (114.3875E,30.6475N) | 1.358973 | 0.922863 |
| (114.4025E,30.4975N) | 1.354613 | 0.919902 |
| (114.4025E,30.5125N) | 1.464789 | 0.994721 |
| (114.4025E,30.6025N) | 1.458588 | 0.99051 |
| (114.4025E,30.6175N) | 1.41966 | 0.964075 |
| (114.4025E,30.6325N) | 1.353225 | 0.918959 |
| (114.4025E,30.6475N) | 1.354183 | 0.91961 |
| (114.4175E,30.5125N) | 1.405319 | 0.954336 |
| (114.4175E,30.6025N) | 1.409313 | 0.957048 |
| (114.4175E,30.6175N) | 1.648884 | 1.119738 |
| (114.4175E,30.6325N) | 1.350201 | 0.916906 |
| (114.4175E,30.6475N) | 1.384915 | 0.940479 |
| (114.4325E,30.6025N) | 1.58779 | 1.07825 |
| (114.4325E,30.6175N) | 1.456033 | 0.988775 |
| (114.4325E,30.6325N) | 1.508925 | 1.024693 |
| (114.4475E,30.6025N) | 1.451527 | 0.985715 |
| (114.4475E,30.6175N) | 1.384931 | 0.94049 |

**Table S3.** $\bar{\boldsymbol{L}_{\boldsymbol{l}}}$ **and** $\boldsymbol{A}_{\bar{\boldsymbol{li}}}$ **values for all nodes in Wuhan in 2023，presented in Fig 8，11.**

| **Coordinates of the Central**  **Point of nodes** | $\bar{\boldsymbol{L}_{\boldsymbol{l}}}$ **Values** | $\boldsymbol{A}_{\bar{\boldsymbol{li}}}$ **Values** |
| --- | --- | --- |
| (114.0875E,30.4525N) | 1.336823 | 0.911119 |
| (114.0875E,30.4675N) | 1.37481 | 0.937009 |
| (114.0875E,30.6025N) | 1.414849 | 0.964298 |
| (114.0875E,30.6175N) | 1.339025 | 0.912619 |
| (114.1025E,30.4525N) | 1.373443 | 0.936077 |
| (114.1025E,30.4675N) | 1.363981 | 0.929629 |
| (114.1025E,30.5425N) | 1.375374 | 0.937393 |
| (114.1025E,30.5575N) | 1.414527 | 0.964078 |
| (114.1025E,30.5875N) | 1.453113 | 0.990377 |
| (114.1025E,30.6025N) | 1.445228 | 0.985003 |
| (114.1025E,30.6175N) | 1.380143 | 0.940644 |
| (114.1025E,30.6325N) | 1.37359 | 0.936177 |
| (114.1175E,30.4525N) | 1.396738 | 0.951954 |
| (114.1175E,30.4675N) | 1.350867 | 0.92069 |
| (114.1175E,30.4825N) | 1.392013 | 0.948734 |
| (114.1175E,30.5425N) | 1.531485 | 1.043791 |
| (114.1175E,30.5575N) | 1.362027 | 0.928297 |
| (114.1175E,30.5725N) | 1.431018 | 0.975318 |
| (114.1175E,30.5875N) | 1.5202 | 1.0361 |
| (114.1175E,30.6025N) | 1.539621 | 1.049337 |
| (114.1175E,30.6175N) | 1.416742 | 0.965588 |
| (114.1175E,30.6325N) | 1.433668 | 0.977124 |
| (114.1175E,30.6475N) | 1.413054 | 0.963075 |
| (114.1325E,30.3925N) | 1.433233 | 0.976827 |
| (114.1325E,30.4075N) | 1.418406 | 0.966722 |
| (114.1325E,30.4225N) | 1.574464 | 1.073084 |
| (114.1325E,30.4375N) | 1.520755 | 1.036479 |
| (114.1325E,30.4675N) | 1.399869 | 0.954088 |
| (114.1325E,30.4825N) | 1.394277 | 0.950277 |
| (114.1325E,30.5425N) | 1.482155 | 1.01017 |
| (114.1325E,30.5575N) | 1.478284 | 1.007532 |
| (114.1325E,30.5725N) | 1.383504 | 0.942934 |
| (114.1325E,30.5875N) | 1.388054 | 0.946035 |
| (114.1325E,30.6025N) | 1.468879 | 1.001122 |
| (114.1325E,30.6175N) | 1.447877 | 0.986808 |
| (114.1325E,30.6325N) | 1.445221 | 0.984998 |
| (114.1325E,30.6475N) | 1.428802 | 0.973807 |
| (114.1325E,30.6625N) | 1.450438 | 0.988553 |
| (114.1475E,30.3925N) | 1.454821 | 0.991541 |
| (114.1475E,30.4075N) | 1.425776 | 0.971745 |
| (114.1475E,30.4225N) | 1.587933 | 1.082264 |
| (114.1475E,30.4375N) | 1.591941 | 1.084996 |
| (114.1475E,30.4525N) | 1.471877 | 1.003166 |
| (114.1475E,30.4675N) | 1.487524 | 1.01383 |
| (114.1475E,30.4825N) | 1.392953 | 0.949375 |
| (114.1475E,30.4975N) | 1.415539 | 0.964768 |
| (114.1475E,30.5125N) | 1.403422 | 0.956509 |
| (114.1475E,30.5275N) | 1.394774 | 0.950615 |
| (114.1475E,30.5425N) | 1.484085 | 1.011486 |
| (114.1475E,30.5575N) | 1.361847 | 0.928174 |
| (114.1475E,30.5725N) | 1.42327 | 0.970037 |
| (114.1475E,30.5875N) | 1.424433 | 0.97083 |
| (114.1475E,30.6025N) | 1.495515 | 1.019276 |
| (114.1475E,30.6175N) | 1.416696 | 0.965557 |
| (114.1475E,30.6325N) | 1.500339 | 1.022564 |
| (114.1475E,30.6475N) | 1.459236 | 0.99455 |
| (114.1475E,30.6625N) | 1.536549 | 1.047243 |
| (114.1475E,30.6775N) | 1.584011 | 1.079591 |
| (114.1625E,30.4075N) | 1.489795 | 1.015378 |
| (114.1625E,30.4225N) | 1.560337 | 1.063456 |
| (114.1625E,30.4375N) | 1.509684 | 1.028933 |
| (114.1625E,30.4525N) | 1.545387 | 1.053266 |
| (114.1625E,30.4825N) | 1.448649 | 0.987334 |
| (114.1625E,30.4975N) | 1.420537 | 0.968175 |
| (114.1625E,30.5125N) | 1.455873 | 0.992258 |
| (114.1625E,30.5425N) | 1.526575 | 1.040445 |
| (114.1625E,30.5575N) | 1.432057 | 0.976026 |
| (114.1625E,30.5725N) | 1.410063 | 0.961036 |
| (114.1625E,30.5875N) | 1.519407 | 1.03556 |
| (114.1625E,30.6025N) | 1.518887 | 1.035205 |
| (114.1625E,30.6175N) | 1.396426 | 0.951742 |
| (114.1625E,30.6325N) | 1.489184 | 1.014961 |
| (114.1625E,30.6625N) | 1.500641 | 1.022769 |
| (114.1625E,30.6775N) | 1.586635 | 1.081379 |
| (114.1625E,30.6925N) | 1.532737 | 1.044645 |
| (114.1775E,30.3925N) | 1.62497 | 1.107507 |
| (114.1775E,30.4375N) | 1.657007 | 1.129342 |
| (114.1775E,30.4525N) | 1.585965 | 1.080923 |
| (114.1775E,30.4675N) | 1.490247 | 1.015686 |
| (114.1775E,30.4825N) | 1.51332 | 1.031411 |
| (114.1775E,30.4975N) | 1.489173 | 1.014954 |
| (114.1775E,30.5125N) | 1.421215 | 0.968637 |
| (114.1775E,30.5275N) | 1.407341 | 0.959181 |
| (114.1775E,30.5425N) | 1.360434 | 0.927211 |
| (114.1775E,30.5725N) | 1.454254 | 0.991154 |
| (114.1775E,30.5875N) | 1.435514 | 0.978382 |
| (114.1775E,30.6025N) | 1.430939 | 0.975264 |
| (114.1775E,30.6175N) | 1.463936 | 0.997753 |
| (114.1775E,30.6325N) | 1.469238 | 1.001367 |
| (114.1775E,30.6625N) | 1.535607 | 1.046601 |
| (114.1775E,30.6775N) | 1.512285 | 1.030706 |
| (114.1775E,30.6925N) | 1.477993 | 1.007334 |
| (114.1925E,30.3925N) | 1.620846 | 1.104696 |
| (114.1925E,30.4075N) | 1.571853 | 1.071305 |
| (114.1925E,30.4225N) | 1.561485 | 1.064238 |
| (114.1925E,30.4525N) | 1.703982 | 1.161358 |
| (114.1925E,30.4675N) | 1.519199 | 1.035418 |
| (114.1925E,30.4825N) | 1.456081 | 0.992399 |
| (114.1925E,30.5125N) | 1.422991 | 0.969847 |
| (114.1925E,30.5275N) | 1.438361 | 0.980322 |
| (114.1925E,30.5425N) | 1.378341 | 0.939415 |
| (114.1925E,30.5725N) | 1.3721 | 0.935162 |
| (114.1925E,30.5875N) | 1.472871 | 1.003843 |
| (114.1925E,30.6025N) | 1.40928 | 0.960502 |
| (114.1925E,30.6175N) | 1.443484 | 0.983814 |
| (114.1925E,30.6325N) | 1.471136 | 1.00266 |
| (114.1925E,30.6475N) | 1.414972 | 0.964382 |
| (114.1925E,30.6775N) | 1.463605 | 0.997528 |
| (114.1925E,30.6925N) | 1.446336 | 0.985758 |
| (114.2075E,30.3925N) | 1.538591 | 1.048634 |
| (114.2075E,30.4075N) | 1.493629 | 1.01799 |
| (114.2075E,30.4225N) | 1.601489 | 1.091503 |
| (114.2075E,30.4375N) | 1.526841 | 1.040627 |
| (114.2075E,30.4675N) | 1.489122 | 1.014919 |
| (114.2075E,30.4825N) | 1.579461 | 1.07649 |
| (114.2075E,30.4975N) | 1.462408 | 0.996712 |
| (114.2075E,30.5125N) | 1.436282 | 0.978906 |
| (114.2075E,30.5275N) | 1.440209 | 0.981582 |
| (114.2075E,30.5425N) | 1.429061 | 0.973984 |
| (114.2075E,30.5575N) | 1.400918 | 0.954803 |
| (114.2075E,30.5725N) | 1.405811 | 0.958138 |
| (114.2075E,30.5875N) | 1.43187 | 0.975898 |
| (114.2075E,30.6025N) | 1.393262 | 0.949585 |
| (114.2075E,30.6175N) | 1.444972 | 0.984828 |
| (114.2075E,30.6325N) | 1.517435 | 1.034216 |
| (114.2075E,30.6475N) | 1.487918 | 1.014098 |
| (114.2075E,30.6625N) | 1.442712 | 0.983288 |
| (114.2075E,30.6775N) | 1.41737 | 0.966016 |
| (114.2225E,30.3925N) | 1.737825 | 1.184424 |
| (114.2225E,30.4075N) | 1.578779 | 1.076025 |
| (114.2225E,30.4225N) | 1.59692 | 1.088389 |
| (114.2225E,30.4375N) | 1.543864 | 1.052229 |
| (114.2225E,30.4525N) | 1.485547 | 1.012482 |
| (114.2225E,30.4825N) | 1.46327 | 0.997299 |
| (114.2225E,30.4975N) | 1.531299 | 1.043665 |
| (114.2225E,30.5125N) | 1.42676 | 0.972415 |
| (114.2225E,30.5275N) | 1.482184 | 1.01019 |
| (114.2225E,30.5575N) | 1.410423 | 0.961281 |
| (114.2225E,30.5725N) | 1.443518 | 0.983837 |
| (114.2225E,30.5875N) | 1.431155 | 0.975411 |
| (114.2225E,30.6025N) | 1.477606 | 1.00707 |
| (114.2225E,30.6175N) | 1.55053 | 1.056772 |
| (114.2225E,30.6325N) | 1.423015 | 0.969863 |
| (114.2225E,30.6475N) | 1.383324 | 0.942811 |
| (114.2225E,30.6625N) | 1.407318 | 0.959165 |
| (114.2225E,30.6775N) | 1.434996 | 0.978029 |
| (114.2375E,30.3925N) | 1.713742 | 1.16801 |
| (114.2375E,30.4225N) | 1.748772 | 1.191884 |
| (114.2375E,30.4375N) | 1.565734 | 1.067134 |
| (114.2375E,30.4525N) | 1.490871 | 1.016111 |
| (114.2375E,30.4675N) | 1.45773 | 0.993524 |
| (114.2375E,30.4975N) | 1.561068 | 1.063954 |
| (114.2375E,30.5125N) | 1.49139 | 1.016464 |
| (114.2375E,30.5275N) | 1.435986 | 0.978704 |
| (114.2375E,30.5575N) | 1.407119 | 0.959029 |
| (114.2375E,30.5725N) | 1.430991 | 0.975299 |
| (114.2375E,30.5875N) | 1.518507 | 1.034947 |
| (114.2375E,30.6025N) | 1.488559 | 1.014535 |
| (114.2375E,30.6175N) | 1.472744 | 1.003757 |
| (114.2375E,30.6325N) | 1.383259 | 0.942768 |
| (114.2375E,30.6475N) | 1.399738 | 0.953999 |
| (114.2375E,30.6625N) | 1.373703 | 0.936254 |
| (114.2525E,30.3925N) | 1.572418 | 1.07169 |
| (114.2525E,30.4075N) | 1.652265 | 1.12611 |
| (114.2525E,30.4225N) | 1.614091 | 1.100092 |
| (114.2525E,30.4375N) | 1.568599 | 1.069087 |
| (114.2525E,30.4525N) | 1.558888 | 1.062468 |
| (114.2525E,30.4675N) | 1.460544 | 0.995441 |
| (114.2525E,30.4825N) | 1.416778 | 0.965612 |
| (114.2525E,30.5125N) | 1.503994 | 1.025055 |
| (114.2525E,30.5275N) | 1.497523 | 1.020644 |
| (114.2525E,30.5425N) | 1.419364 | 0.967375 |
| (114.2525E,30.5575N) | 1.381078 | 0.941281 |
| (114.2525E,30.5725N) | 1.427762 | 0.973099 |
| (114.2525E,30.5875N) | 1.453735 | 0.990801 |
| (114.2525E,30.6025N) | 1.443403 | 0.983759 |
| (114.2525E,30.6175N) | 1.382984 | 0.94258 |
| (114.2525E,30.6325N) | 1.396689 | 0.951921 |
| (114.2525E,30.6475N) | 1.407277 | 0.959137 |
| (114.2525E,30.6625N) | 1.473689 | 1.0044 |
| (114.2525E,30.6775N) | 1.577884 | 1.075415 |
| (114.2675E,30.3925N) | 1.547606 | 1.054779 |
| (114.2675E,30.4075N) | 1.572677 | 1.071866 |
| (114.2675E,30.4225N) | 1.6205 | 1.10446 |
| (114.2675E,30.4375N) | 1.56559 | 1.067036 |
| (114.2675E,30.4525N) | 1.480726 | 1.009196 |
| (114.2675E,30.4675N) | 1.569339 | 1.069591 |
| (114.2675E,30.4825N) | 1.436517 | 0.979066 |
| (114.2675E,30.4975N) | 1.498184 | 1.021095 |
| (114.2675E,30.5425N) | 1.562328 | 1.064812 |
| (114.2675E,30.5575N) | 1.5261 | 1.040122 |
| (114.2675E,30.5725N) | 1.382111 | 0.941985 |
| (114.2675E,30.5875N) | 1.428057 | 0.9733 |
| (114.2675E,30.6025N) | 1.425747 | 0.971725 |
| (114.2675E,30.6175N) | 1.446906 | 0.986147 |
| (114.2675E,30.6325N) | 1.422188 | 0.9693 |
| (114.2675E,30.6475N) | 1.386282 | 0.944827 |
| (114.2675E,30.6625N) | 1.509223 | 1.028619 |
| (114.2675E,30.6775N) | 1.408073 | 0.959679 |
| (114.2675E,30.6925N) | 1.421892 | 0.969098 |
| (114.2825E,30.3925N) | 1.522119 | 1.037408 |
| (114.2825E,30.4075N) | 1.508084 | 1.027842 |
| (114.2825E,30.4675N) | 1.42428 | 0.970725 |
| (114.2825E,30.4825N) | 1.487741 | 1.013978 |
| (114.2825E,30.4975N) | 1.429159 | 0.974051 |
| (114.2825E,30.5125N) | 1.535158 | 1.046295 |
| (114.2825E,30.5575N) | 1.632116 | 1.112377 |
| (114.2825E,30.5725N) | 1.59848 | 1.089452 |
| (114.2825E,30.5875N) | 1.451472 | 0.989258 |
| (114.2825E,30.6025N) | 1.415563 | 0.964784 |
| (114.2825E,30.6175N) | 1.389805 | 0.947229 |
| (114.2825E,30.6325N) | 1.388016 | 0.946009 |
| (114.2825E,30.6475N) | 1.416714 | 0.965569 |
| (114.2825E,30.6625N) | 1.420706 | 0.96829 |
| (114.2825E,30.6775N) | 1.421551 | 0.968865 |
| (114.2825E,30.6925N) | 1.447533 | 0.986573 |
| (114.2975E,30.3925N) | 1.413736 | 0.963539 |
| (114.2975E,30.4075N) | 1.452562 | 0.990001 |
| (114.2975E,30.4225N) | 1.466985 | 0.999831 |
| (114.2975E,30.4375N) | 1.471205 | 1.002707 |
| (114.2975E,30.4525N) | 1.436972 | 0.979375 |
| (114.2975E,30.4675N) | 1.464514 | 0.998147 |
| (114.2975E,30.4825N) | 1.520291 | 1.036162 |
| (114.2975E,30.4975N) | 1.542051 | 1.050993 |
| (114.2975E,30.5125N) | 1.49278 | 1.017412 |
| (114.2975E,30.5275N) | 1.447156 | 0.986317 |
| (114.2975E,30.5425N) | 1.499074 | 1.021701 |
| (114.2975E,30.5575N) | 1.580945 | 1.077501 |
| (114.2975E,30.5875N) | 1.499736 | 1.022153 |
| (114.2975E,30.6025N) | 1.394343 | 0.950322 |
| (114.2975E,30.6175N) | 1.420683 | 0.968274 |
| (114.2975E,30.6325N) | 1.447225 | 0.986364 |
| (114.2975E,30.6475N) | 1.45237 | 0.98987 |
| (114.2975E,30.6625N) | 1.418955 | 0.967096 |
| (114.2975E,30.6775N) | 1.511775 | 1.030358 |
| (114.3125E,30.3925N) | 1.403661 | 0.956673 |
| (114.3125E,30.4075N) | 1.427357 | 0.972823 |
| (114.3125E,30.4225N) | 1.449707 | 0.988055 |
| (114.3125E,30.4375N) | 1.423097 | 0.969919 |
| (114.3125E,30.4525N) | 1.44049 | 0.981773 |
| (114.3125E,30.4675N) | 1.471921 | 1.003196 |
| (114.3125E,30.4825N) | 1.561939 | 1.064548 |
| (114.3125E,30.4975N) | 1.4837 | 1.011223 |
| (114.3125E,30.5125N) | 1.472681 | 1.003714 |
| (114.3125E,30.5275N) | 1.447516 | 0.986562 |
| (114.3125E,30.5425N) | 1.437951 | 0.980043 |
| (114.3125E,30.5575N) | 1.418532 | 0.966808 |
| (114.3125E,30.5725N) | 1.498 | 1.02097 |
| (114.3125E,30.6025N) | 1.467655 | 1.000288 |
| (114.3125E,30.6175N) | 1.422152 | 0.969275 |
| (114.3125E,30.6325N) | 1.379178 | 0.939986 |
| (114.3125E,30.6475N) | 1.387791 | 0.945856 |
| (114.3125E,30.6625N) | 1.358304 | 0.925759 |
| (114.3125E,30.6775N) | 1.449888 | 0.988178 |
| (114.3275E,30.4075N) | 1.470438 | 1.002185 |
| (114.3275E,30.4225N) | 1.544716 | 1.052809 |
| (114.3275E,30.4375N) | 1.488704 | 1.014634 |
| (114.3275E,30.4675N) | 1.49461 | 1.018659 |
| (114.3275E,30.4825N) | 1.491248 | 1.016368 |
| (114.3275E,30.4975N) | 1.451325 | 0.989158 |
| (114.3275E,30.5125N) | 1.448262 | 0.98707 |
| (114.3275E,30.5275N) | 1.428176 | 0.97338 |
| (114.3275E,30.5425N) | 1.399182 | 0.95362 |
| (114.3275E,30.5575N) | 1.460323 | 0.995291 |
| (114.3275E,30.5725N) | 1.422783 | 0.969705 |
| (114.3275E,30.5875N) | 1.434726 | 0.977845 |
| (114.3275E,30.6025N) | 1.444369 | 0.984417 |
| (114.3275E,30.6325N) | 1.425326 | 0.971439 |
| (114.3275E,30.6475N) | 1.39121 | 0.948187 |
| (114.3275E,30.6625N) | 1.380546 | 0.940918 |
| (114.3275E,30.6775N) | 1.346543 | 0.917744 |
| (114.3425E,30.4525N) | 1.484459 | 1.011741 |
| (114.3425E,30.4675N) | 1.488986 | 1.014826 |
| (114.3425E,30.4825N) | 1.535134 | 1.046279 |
| (114.3425E,30.4975N) | 1.435108 | 0.978105 |
| (114.3425E,30.5125N) | 1.453203 | 0.990438 |
| (114.3425E,30.5275N) | 1.434036 | 0.977374 |
| (114.3425E,30.5425N) | 1.419069 | 0.967174 |
| (114.3425E,30.5575N) | 1.409767 | 0.960834 |
| (114.3425E,30.5725N) | 1.463071 | 0.997163 |
| (114.3425E,30.5875N) | 1.413604 | 0.963449 |
| (114.3425E,30.6025N) | 1.382604 | 0.942321 |
| (114.3425E,30.6175N) | 1.419415 | 0.96741 |
| (114.3425E,30.6475N) | 1.544325 | 1.052542 |
| (114.3425E,30.6625N) | 1.364153 | 0.929746 |
| (114.3425E,30.6775N) | 1.354357 | 0.923069 |
| (114.3575E,30.3925N) | 1.49488 | 1.018843 |
| (114.3575E,30.4075N) | 1.564727 | 1.066448 |
| (114.3575E,30.4525N) | 1.493007 | 1.017567 |
| (114.3575E,30.4675N) | 1.591728 | 1.084851 |
| (114.3575E,30.4825N) | 1.553967 | 1.059114 |
| (114.3575E,30.4975N) | 1.433955 | 0.97732 |
| (114.3575E,30.5125N) | 1.515139 | 1.032651 |
| (114.3575E,30.5275N) | 1.426209 | 0.97204 |
| (114.3575E,30.5425N) | 1.427053 | 0.972615 |
| (114.3575E,30.5575N) | 1.552748 | 1.058283 |
| (114.3575E,30.5725N) | 1.419166 | 0.96724 |
| (114.3575E,30.5875N) | 1.37657 | 0.938209 |
| (114.3575E,30.6025N) | 1.354157 | 0.922933 |
| (114.3575E,30.6175N) | 1.545449 | 1.053309 |
| (114.3575E,30.6325N) | 1.353797 | 0.922687 |
| (114.3725E,30.3775N) | 1.49236 | 1.017126 |
| (114.3725E,30.3925N) | 1.51969 | 1.035752 |
| (114.3725E,30.4075N) | 1.509411 | 1.028747 |
| (114.3725E,30.4225N) | 1.492256 | 1.017054 |
| (114.3725E,30.4525N) | 1.548925 | 1.055678 |
| (114.3725E,30.4675N) | 1.517059 | 1.033959 |
| (114.3725E,30.4825N) | 1.46136 | 0.995998 |
| (114.3725E,30.4975N) | 1.511455 | 1.03014 |
| (114.3725E,30.5125N) | 1.404969 | 0.957564 |
| (114.3725E,30.5275N) | 1.470237 | 1.002048 |
| (114.3725E,30.5425N) | 1.444553 | 0.984542 |
| (114.3725E,30.5575N) | 1.467637 | 1.000276 |
| (114.3725E,30.5725N) | 1.421786 | 0.969026 |
| (114.3725E,30.5875N) | 1.432948 | 0.976633 |
| (114.3725E,30.6025N) | 1.389065 | 0.946725 |
| (114.3725E,30.6175N) | 1.371543 | 0.934782 |
| (114.3725E,30.6325N) | 1.367829 | 0.932251 |
| (114.3875E,30.3775N) | 1.545027 | 1.053021 |
| (114.3875E,30.4375N) | 1.464802 | 0.998343 |
| (114.3875E,30.4525N) | 1.439162 | 0.980868 |
| (114.3875E,30.4675N) | 1.445789 | 0.985385 |
| (114.3875E,30.4825N) | 1.44351 | 0.983832 |
| (114.3875E,30.4975N) | 1.41185 | 0.962254 |
| (114.3875E,30.5125N) | 1.484849 | 1.012007 |
| (114.3875E,30.5275N) | 1.500373 | 1.022587 |
| (114.3875E,30.5875N) | 1.423406 | 0.97013 |
| (114.3875E,30.6025N) | 1.432053 | 0.976023 |
| (114.3875E,30.6175N) | 1.399037 | 0.953521 |
| (114.3875E,30.6325N) | 1.337447 | 0.911544 |
| (114.3875E,30.6475N) | 1.331481 | 0.907478 |
| (114.4025E,30.3775N) | 1.489838 | 1.015407 |
| (114.4025E,30.3925N) | 1.53595 | 1.046834 |
| (114.4025E,30.4075N) | 1.634454 | 1.11397 |
| (114.4025E,30.4225N) | 1.479173 | 1.008138 |
| (114.4025E,30.4525N) | 1.44345 | 0.983791 |
| (114.4025E,30.4675N) | 1.430227 | 0.974779 |
| (114.4025E,30.4825N) | 1.379246 | 0.940032 |
| (114.4025E,30.4975N) | 1.363427 | 0.929251 |
| (114.4025E,30.5125N) | 1.459519 | 0.994743 |
| (114.4025E,30.5275N) | 1.512871 | 1.031105 |
| (114.4025E,30.5425N) | 1.598787 | 1.089661 |
| (114.4025E,30.6025N) | 1.427582 | 0.972976 |
| (114.4025E,30.6175N) | 1.375502 | 0.937481 |
| (114.4025E,30.6325N) | 1.333217 | 0.908661 |
| (114.4025E,30.6475N) | 1.312545 | 0.894572 |
| (114.4175E,30.3775N) | 1.50743 | 1.027397 |
| (114.4175E,30.3925N) | 1.476828 | 1.00654 |
| (114.4175E,30.4075N) | 1.445446 | 0.985151 |
| (114.4175E,30.4225N) | 1.479707 | 1.008502 |
| (114.4175E,30.4375N) | 1.426311 | 0.97211 |
| (114.4175E,30.4525N) | 1.38059 | 0.940949 |
| (114.4175E,30.4675N) | 1.545097 | 1.053069 |
| (114.4175E,30.4825N) | 1.372103 | 0.935164 |
| (114.4175E,30.4975N) | 1.522746 | 1.037835 |
| (114.4175E,30.5125N) | 1.418778 | 0.966975 |
| (114.4175E,30.5275N) | 1.477819 | 1.007215 |
| (114.4175E,30.5425N) | 1.813263 | 1.235839 |
| (114.4175E,30.5725N) | 1.748994 | 1.192036 |
| (114.4175E,30.5875N) | 1.690627 | 1.152255 |
| (114.4175E,30.6025N) | 1.371913 | 0.935035 |
| (114.4175E,30.6175N) | 1.461513 | 0.996102 |
| (114.4175E,30.6325N) | 1.330846 | 0.907045 |
| (114.4175E,30.6475N) | 1.350644 | 0.920538 |
| (114.4325E,30.3925N) | 1.453075 | 0.990351 |
| (114.4325E,30.4075N) | 1.440416 | 0.981723 |
| (114.4325E,30.4225N) | 1.422114 | 0.969249 |
| (114.4325E,30.4375N) | 1.412207 | 0.962497 |
| (114.4325E,30.4525N) | 1.378148 | 0.939284 |
| (114.4325E,30.4675N) | 1.382011 | 0.941917 |
| (114.4325E,30.4825N) | 1.342225 | 0.9148 |
| (114.4325E,30.4975N) | 1.392291 | 0.948923 |
| (114.4325E,30.5125N) | 1.404638 | 0.957339 |
| (114.4325E,30.5275N) | 1.508262 | 1.027964 |
| (114.4325E,30.5725N) | 1.670108 | 1.138271 |
| (114.4325E,30.5875N) | 1.740061 | 1.185948 |
| (114.4325E,30.6025N) | 1.49142 | 1.016485 |
| (114.4325E,30.6175N) | 1.410656 | 0.96144 |
| (114.4325E,30.6325N) | 1.467052 | 0.999877 |
| (114.4475E,30.4075N) | 1.546078 | 1.053738 |
| (114.4475E,30.4225N) | 1.364199 | 0.929777 |
| (114.4475E,30.4375N) | 1.404256 | 0.957078 |
| (114.4475E,30.4525N) | 1.402147 | 0.955641 |
| (114.4475E,30.4675N) | 1.387665 | 0.94577 |
| (114.4475E,30.4825N) | 1.354792 | 0.923366 |
| (114.4475E,30.4975N) | 1.355228 | 0.923663 |
| (114.4475E,30.5125N) | 1.44703 | 0.98623 |
| (114.4475E,30.5275N) | 1.633005 | 1.112983 |
| (114.4475E,30.5575N) | 1.525953 | 1.040021 |
| (114.4475E,30.5725N) | 1.623577 | 1.106557 |
| (114.4475E,30.5875N) | 1.557458 | 1.061494 |
| (114.4475E,30.6025N) | 1.518257 | 1.034776 |
| (114.4475E,30.6175N) | 1.417484 | 0.966093 |
| (114.4625E,30.4225N) | 1.397389 | 0.952398 |
| (114.4625E,30.4375N) | 1.380029 | 0.940566 |
| (114.4625E,30.4525N) | 1.375148 | 0.937239 |
| (114.4625E,30.4675N) | 1.389105 | 0.946752 |
| (114.4625E,30.4825N) | 1.418044 | 0.966476 |
| (114.4625E,30.4975N) | 1.496723 | 1.020099 |
| (114.4625E,30.5125N) | 1.384728 | 0.943769 |
| (114.4625E,30.5275N) | 1.453594 | 0.990705 |
| (114.4625E,30.5425N) | 1.535761 | 1.046706 |
| (114.4625E,30.5575N) | 1.552522 | 1.05813 |
| (114.4625E,30.5875N) | 1.557598 | 1.061589 |
| (114.4625E,30.6025N) | 1.505561 | 1.026123 |
| (114.4625E,30.6175N) | 1.452617 | 0.990039 |
| (114.4775E,30.4675N) | 1.366542 | 0.931373 |
| (114.4775E,30.4825N) | 1.157558 | 0.788939 |
| (114.4775E,30.4975N) | 1.406194 | 0.958398 |
| (114.4775E,30.5125N) | 1.399507 | 0.953841 |
| (114.4775E,30.5275N) | 1.584401 | 1.079857 |
| (114.4775E,30.5875N) | 1.612415 | 1.09895 |
| (114.4775E,30.6025N) | 1.521102 | 1.036715 |
| (114.4775E,30.6175N) | 1.549375 | 1.055985 |
| (114.4925E,30.4675N) | 1.400438 | 0.954476 |
| (114.4925E,30.4825N) | 1.277611 | 0.870762 |
| (114.4925E,30.4975N) | 1.302967 | 0.888044 |
| (114.4925E,30.5125N) | 1.372796 | 0.935636 |
| (114.4925E,30.5275N) | 1.552412 | 1.058054 |
| (114.4925E,30.5425N) | 1.628775 | 1.1101 |
| (114.4925E,30.5575N) | 1.545666 | 1.053456 |
| (114.4925E,30.5725N) | 1.571669 | 1.071179 |
| (114.4925E,30.5875N) | 1.776666 | 1.210896 |
| (114.4925E,30.6025N) | 1.637651 | 1.116149 |
| (114.5075E,30.4675N) | 1.374174 | 0.936576 |
| (114.5075E,30.4825N) | 1.350051 | 0.920135 |
| (114.5075E,30.4975N) | 1.337557 | 0.911619 |
| (114.5075E,30.5125N) | 1.366373 | 0.931259 |
| (114.5075E,30.5275N) | 1.49917 | 1.021767 |
| (114.5075E,30.5425N) | 1.506398 | 1.026694 |
| (114.5075E,30.5575N) | 1.492456 | 1.017191 |
| (114.5075E,30.5725N) | 1.546786 | 1.05422 |
| (114.5075E,30.6025N) | 1.749143 | 1.192137 |
| (114.5225E,30.4525N) | 1.474125 | 1.004697 |
| (114.5225E,30.4675N) | 1.453768 | 0.990823 |
| (114.5225E,30.4825N) | 1.328084 | 0.905163 |
| (114.5225E,30.4975N) | 1.301033 | 0.886725 |
| (114.5225E,30.5125N) | 1.3942 | 0.950224 |
| (114.5225E,30.6025N) | 1.789291 | 1.219501 |
| (114.5375E,30.4525N) | 1.390858 | 0.947946 |
| (114.5375E,30.4675N) | 1.441349 | 0.982359 |
| (114.5375E,30.4825N) | 1.347359 | 0.9183 |
| (114.5375E,30.4975N) | 1.329446 | 0.906091 |
| (114.5525E,30.4525N) | 1.401989 | 0.955533 |
| (114.5525E,30.4675N) | 1.426064 | 0.971941 |
